# Spatio-temporal dynamics, transmission hotspots, and vaccination gaps driving measles persistence in Nigeria, 2006–2025

**DOI:** 10.1371/journal.pone.0359837

**Published:** 2026-10-27

**Authors:** Sylvia Ezenwa-Ahanene, Polycarp Dauda Madaki, Ruth Manzo Sabo, Oghenetega ThankGod Oweh, Kalat Haruna Tauna, Zubairu Zubairu Dalhatu, Mustapha Sikirat Abiola, Ifeoma Christiana Nwadiuto, Muzzammil Gadanya, Abubakar Yahya Imam

**Affiliations:** 1 Nigeria Centre for Disease Control and Prevention, Abuja, Nigeria; 2 Corona Management Systems, Kado, Abuja, Nigeria; 3 Department of Veterinary Tropical Diseases, University of Pretoria, Pretoria, South Africa; 4 African Centre for Disease Control and Prevention, Addis Ababa, Ethiopia; 5 Department of Medical Biochemistry, College of Medicine, Kaduna State University, Kaduna, Nigeria; 6 Department of Public Health, Faculty of Basic and Applied Biological Sciences, Ahmadu Bello University, Zaria, Nigeria; 7 Kwara State Ministry of Health, Ilorin, Nigeria; 8 Department of Community Medicine, Rivers State University, Rivers, Nigeria; 9 National Primary Healthcare Development Agency, Abuja, Nigeria; Universitas Syiah Kuala, INDONESIA

## Abstract

**Background:**

Measles remains a major public health concern in Nigeria, contributing disproportionately to the global burden. Persistence of the disease is driven by sub-optimal vaccination coverage and heterogeneous transmission patterns, but the specific spatio-temporal dynamics and the quantitative impact of vaccination gaps at the sub-national level are not fully characterized.

**Objective:**

This study aimed to provide a comprehensive analysis of measles transmission in Nigeria from 2006 to 2025, with the specific objectives to identify spatial hotspots, quantify their temporal persistence, and evaluate the association between MCV1/MCV2 coverage and measles case ratio.

**Methods:**

We conducted a national retrospective ecological study, integrating 19 years of surveillance data (2006–2025) and geospatial information, with a focused 4-year analysis of vaccination records (2021–2024). Analytical methods included time-series decomposition, spatial autocorrelation (Local Moran’s I and Getis-Ord Gi*), and linear mixed-effects modeling to quantify vaccination effects while accounting for state-level heterogeneity.

**Results:**

Analysis of 253,595 measles cases revealed a highly clustered geographic distribution, with 86.4% of the national burden concentrated in northern Nigeria. We identified 38 persistent hotspot Local Government Areas that accounted for 23.2% of all cases. Vaccination coverage was low and disparate, with national averages of 73.8% for MCV1 and 38.4% for MCV2. Mixed-effects modeling confirmed a significant negative association between MCV1 coverage and the measles case ratio, while MCV2 coverage did not show a statistically significant independent effect.

**Conclusion:**

Measles transmission in Nigeria is driven by entrenched hotspots in the north and critical vaccination gaps threatening disease resurgence in the south. Achieving elimination requires a tailored strategy: intensive supplemental immunization activities and RI strengthening aimed at reducing endemic transmission in the northern hotspots, coupled with focused efforts to close routine immunization gaps in specific southern states.

## 1. Introduction

Measles remains one of the most contagious human pathogens, causing infections in more than 90% of susceptible contacts because its basic reproduction number $R_{\text{0}}$ is estimated to be between 12 and 18 [1–3]. Despite global efforts toward the eradication of measles, many low- and middle-income countries-some in sub-Saharan Africa-continue to experience cyclical outbreaks driven by low routine vaccination coverage, demographic pressures, and spatially heterogeneous transmission patterns [4–6]. Nigeria is the most populated nation in Africa and continues to be a significant burden to global morbidity and mortality of measles. Nigeria represented about 47 percent of the total number of zero-dose children (those who have not received any routine vaccinations) in the world in 2022 and was ranked as one of the top 10 countries that contributed more than half of all measles cases in the world [7,8]. Notwithstanding the progress, outbreaks still take place, which makes Nigeria an important target when it comes to eliminating measles [9].

Although vaccination campaigns against measles have raised the rate of immunization against measles-containing vaccine (MCV), coverage remains below the ≥ 95% threshold required for herd immunity [7]. The presence of transmission reservoirs due to persistent sub-national disparities (especially between northern and southern states) is observed as the MCV1 coverage is as low as 20–31% in the north and 43–57% in the south [10–12]. In 2019, Nigeria implemented measles second-dose vaccination (MCV2) in the routine immunization program, which is focused on closing immunity gaps, yet administrative coverage is much lower and has a high over-reporting rate in comparison with survey estimates [11]. Presence of these gaps predetermines recurrent outbreaks, accumulation of vulnerable groups, and long-term spatial proliferation of the measles outbreaks [6].

It is important to understand the spatial and temporal dynamics of spread of measles to implement specific vaccination intervention. A growing body of literature highlights the value of geospatial analytics including Local Indicators of Spatial Association (LISA), Getis-Ord Gi*, and space time cluster analyses for detecting high-transmission foci, quantifying persistence, and identifying areas where routine immunization and outbreak response require prioritization [13,14]. Nevertheless, there are limited studies which have utilized these tools on long-term measles surveillance data over almost 2 decades on the integration of both administrative vaccination coverage and the finer case records of epidemiologic surveillance.

In addition, the percentage of vaccination and the number of cases of measles are not well estimated at the sub-national level in Nigeria. Although inverse correlations between the coverage of measles-containing vaccine and incidence have been established in some previous studies in Africa [15–17], it is still not known whether MCV1 and MCV2 interact, whether there might be potential immunization thresholds, and what the relative contribution of geographic heterogeneity might be in the case of Nigeria. The identification of these gaps is very critical to achieving the objectives of the World Health Organization on the elimination of measles in the region [7].

This study therefore provides the most comprehensive spatio-temporal analysis of measles transmission in Nigeria to date, integrating 19 years of case surveillance (2006 through the first quarter of 2025), vaccination administrative data, and high-resolution geospatial information. Specifically, we characterize national and sub-national temporal trends (2006–2025); identify spatial clusters and persistent hotspots (2006–2025); and examine associations between MCV1/MCV2 coverage (2021–2024) and measles case ratio using mixed-effects modeling. The findings offer actionable evidence for tailored immunization strategies and priority setting for measles elimination in Nigeria.

## 2. Methods

### 2.1. Study design, data sources, and pre-processing

We conducted a retrospective ecological study of measles transmission dynamics in Nigeria. The analysis of spatio-temporal trends and hotspots utilized measles case data from January 2006 to April 2025. The analysis of vaccination coverage and its association with incidence was restricted to the period of January 2021 to December 2024, for which complete vaccination and live birth data were available.

#### 2.1.1. Data sources and variables.

**Measles Case Data:** Individual-level records of confirmed and probable measles cases (January 2006–April 2025) were obtained from the national measles surveillance system jointly curated by the Nigeria Centre for Disease Control and Prevention (NCDC) and the World Health Organization Regional Office for Africa (WHO AFRO). The dataset included variables such as date of onset, Local Government Area (LGA), case classification, age group, and vaccination status.

**Vaccination and Demographic Data:** Monthly state-level data on MCV1 and MCV2 doses administered, as well as live birth records (January 2021–December 2024), were obtained through the District Health Information System 2 (DHIS2).

**Geospatial Data:** Administrative boundary shapefiles for states and LGAs were acquired from the Database of Global Administrative Areas (GADM, version 4.1).

#### 2.1.2. Data integration and management.

Measles case data underwent extensive cleaning, including duplicate removal and standardization of LGA names using fuzzy-matching algorithms, achieving a 99.9% concordance with GADM reference names. Cases were aggregated into LGA-month units for spatio-temporal processing.

Monthly vaccination coverage for each state was computed using:


$$\begin{array}{c} \text{MCV }{\text{Coverage}}_{s,t}=\left(\frac{\text{MCV Doses }{\text{Administered}}_{s,t}}{\text{Live }{\text{Births}}_{s,t}}\right)\times \text{1}\text{0}\text{0} \end{array}$$

where (s) represents state and (t) represents month.

Similarly, measles case ratio was calculated as:


$$\begin{array}{c} \text{Case }{\text{Ratio}}_{s,t}=\left(\frac{\text{Measles }{\text{Cases}}_{s,t}}{\text{Live }{\text{Births}}_{s,t}}\right)\times \text{1}\text{0}\text{0},\text{0}\text{0}\text{0} \end{array}$$

The case ratio was calculated per 100,000 live births. This approach, while non-standard for general disease burden measurement, was necessary due to the unavailability of reliable sub-national population estimates for the full study period. More importantly, it provides analytical consistency when examining the relationship between vaccination coverage (which uses the birth cohort as its denominator) and disease case ratio. This method has been employed in previous measles studies facing similar data constraints and is particularly relevant for measles, where the birth cohort represents both the primary vaccination target and the most susceptible population [18–21].

All analyses were performed in Microsoft Excel and R (version 4.3.0) using packages including tidyverse, sf, spdep, and lme4.

### 2.2. Spatio-temporal trend and hotspot analysis

#### 2.2.1. Descriptive and temporal analysis.

Case characteristics were summarized using descriptive statistics. National monthly case counts were examined to identify long-term trends and outbreak peaks. A classical multiplicative time-series decomposition was applied to separate trend, seasonal, and irregular components, allowing quantification of seasonal strength.

#### 2.2.2. Spatial autocorrelation and clustering.

Global spatial autocorrelation of cumulative LGA-level measles burden was assessed using Global Moran’s I, based on a queen-contiguity spatial weights matrix. Statistical significance was evaluated using 999 Monte Carlo permutations.

Local Indicators of Spatial Association (LISA) were computed to identify significant local clusters (high-high, low-low) and spatial outliers at 90%, 95%, and 99% confidence levels.

#### 2.2.3. Hotspot and persistence analysis.

The Getis-Ord Gi* statistic was applied using a 6-nearest-neighbors spatial weights matrix. This choice was guided by the need to capture local transmission that may extend beyond immediate administrative boundaries. A 6-neighbor configuration approximates the estimated mean radius of measles case importation and secondary spread in a highly mobile population. Alternative spatial weights, such as queen contiguity and fixed-distance bands, were initially explored; however, the 6-nearest-neighbors matrix provided the most coherent and geographically meaningful identification of contiguous hotspot clusters.

Temporal persistence of high transmission was quantified by computing the fraction of study years during which each LGA fell within the national top 5% of annual case counts:


$$\begin{array}{c} {\text{Persistence}}_{i}=\frac{\text{Number of Years }{\text{LGA}}_{i}\;\text{is in Top }\text{5}\%}{\text{Total Number of Years}} \end{array}$$

The total number of years in the study period for this analysis was 19.

#### 2.2.4. Integrated risk classification.

A multi-criteria risk framework was developed by integrating LISA cluster categories, Gi* hotspot significance, temporal persistence scores, and absolute case burden. LGAs were classified into a four-category risk framework integrating the results of the LISA, Gi*, persistence, and burden analyses. The final categories were: ‘Persistent High-Risk Hotspot’ (significant spatial cluster/hotspot with high persistence), ‘High Case Burden’ (high burden but not a significant spatial cluster), ‘Low Risk’ (low burden and not a cluster), and ‘Spatial Cluster Only’ (significant clustering without high persistence or absolute burden). LGAs were classified as having high persistence if they appeared in the national top 5% of annual case counts in ≥30% of study years. High burden was defined as belonging to the upper quartile (75th percentile) of cumulative measles cases across all LGAs. Persistent High-Risk Hotspots were LGAs meeting criteria for significant spatial clustering (LISA or Gi*), high persistence, and high burden. High Case Burden LGAs met the burden criterion but not hotspot criteria. Spatial Cluster Only LGAs met hotspot criteria without meeting burden or persistence thresholds.

### 2.3. Vaccination coverage and risk factor modeling

#### 2.3.1. Coverage and Incidence Relationships.

National and state-level MCV1 and MCV2 coverage trends were summarized. The initial relationship between vaccination coverage and measles case ratio was assessed using Pearson correlation coefficients.

#### 2.3.2. Mixed-effects regression modeling.

The analysis of vaccination coverage and its association with measles case ratio was conducted on data from January 2021 to December 2024 to ensure temporal alignment of all variables. To model the association between vaccination coverage and measles case ratio while accounting for repeated state-level observations, linear mixed-effects models were fitted using log-transformed case ratio as the outcome:


$$\begin{array}{c} Y_{s,t}=\text{log}\left(\text{Case }{\text{Ratio}}_{s,t}+\text{1}\right) \end{array}$$

Both MCV1 and MCV2 coverage values were standardized via z-transformation. Each model included a random intercept for state to capture unobserved, time-invariant heterogeneity. The nested models were:

- Mixed-Effects Model 1: $Y\sim {\text{MCV1}}_{z}+{\text{MCV2}}_{z}+\left(\text{1}|\text{State}\right)$
- Mixed-Effects Model 2: $Y\sim {\text{MCV1}}_{z}+{\text{MCV2}}_{z}+\text{Time}+\left(\text{1}|\text{State}\right)$
- Mixed-Effects Model 3: $Y\sim {\text{MCV1}}_{z}\times {\text{MCV2}}_{z}+\text{Time}+\left(\text{1}|\text{State}\right)$

where Time is a continuous numeric variable representing secular temporal trends.

### 2.4. Model diagnostics and assumption checking

To ensure the validity of the mixed-effects models, we conducted comprehensive diagnostic assessments. Temporal autocorrelation in the residuals was examined using the Durbin-Watson test. Collinearity between predictors was assessed using the Variance Inflation Factor (VIF), with values below 10 considered acceptable. Residual assumptions were evaluated using the Shapiro-Wilk test for normality, and Cook’s distance was calculated to identify influential observations (values > 0.5 were considered influential). Marginal R² (variance explained by fixed effects) and conditional R² (variance explained by fixed and random effects) were calculated to assess model fit.

#### 2.4.1. Threshold and intervention prioritization analysis.

An outbreak month was defined as any month with case counts exceeding the national 75th percentile. This threshold was chosen as a pragmatic but conservative definition to identify months with higher-than-usual transmission, focusing on the upper quartile of the case distribution. Receiver Operating Characteristic (ROC) analysis was used to evaluate the discriminative performance of MCV1 coverage for identifying outbreak months. Sensitivity, specificity, and the area under the curve (AUC) with 95% confidence intervals were calculated.

### 2.5. Ethics approval

Ethical clearance for the use of the secondary data applied in this study was granted by the National Health Research Ethics Committee (NHREC), under Protocol Number NHREC/01/01/2007–27/10/2023 and Approval Number NHREC/01/01/2007–18/02/2025. All data were fully anonymized prior to analysis, contained no personal identifiers, and could not be used to trace or identify any individual. The anonymized surveillance datasets used for this analysis were accessed in May 2025.

## 3. Results

### 3.1. Descriptive epidemiology of measles cases

A total of 253,595 measles cases were reported in Nigeria from January 2006 to April 2025. The majority of cases were classified as clinically compatible (46.5%, n = 117,915) or epidemiologically linked (39.1%, n = 99,184), with 14.4% (n = 36,496) being laboratory-confirmed (Table 1). A slight male predominance was observed (51.9%). The disease disproportionately affected young children, with the 1–4 years age group accounting for 61.6% (n = 156,178) of all cases. Most cases (68.9%) occurred in individuals with no history of measles vaccination, and only 3.2% had received two doses (Table 1).

**Table 1:** Descriptive summary of measles cases by classification, sex, age, and vaccination status, Nigeria (2006–2025).

Characteristic	Category	Cases	(%)
**Case Classification**	Clinically compatible	117,915	46.5
	Epidemiologically linked	99,184	39.1
	Laboratory-confirmed	36,496	14.4
**Sex**	Male	131,497	51.9
	Female	121,740	48.0
	Unknown	358	0.1
**Age Group**	0–11 months	23,787	9.4
	1–4 years	156,178	61.6
	5–14 years	54,288	21.4
	15 + years	8,520	3.4
	Unknown	10,822	4.3
**Vaccination Status**	No vaccination	174,731	68.9
	1 dose	36,438	14.4
	2 doses	8,096	3.2
	3 doses	1,698	0.7
	Unknown	32,632	12.9

### 3.2. Temporal trends and spatial distribution

National surveillance data revealed substantial temporal fluctuations in measles incidence over the 19-year period. The time series was characterized by significant inter-annual outbreaks, with the most pronounced peak occurring in March 2013, reporting over 15,000 cases in a single month (Fig 1). Time-series decomposition identified a weak but recurrent seasonal pattern, with consistent peaks occurring between December and February, although the seasonal component accounted for a relatively small proportion of the overall variance compared to the trend and residual components (Fig 2).

**Fig 1:**
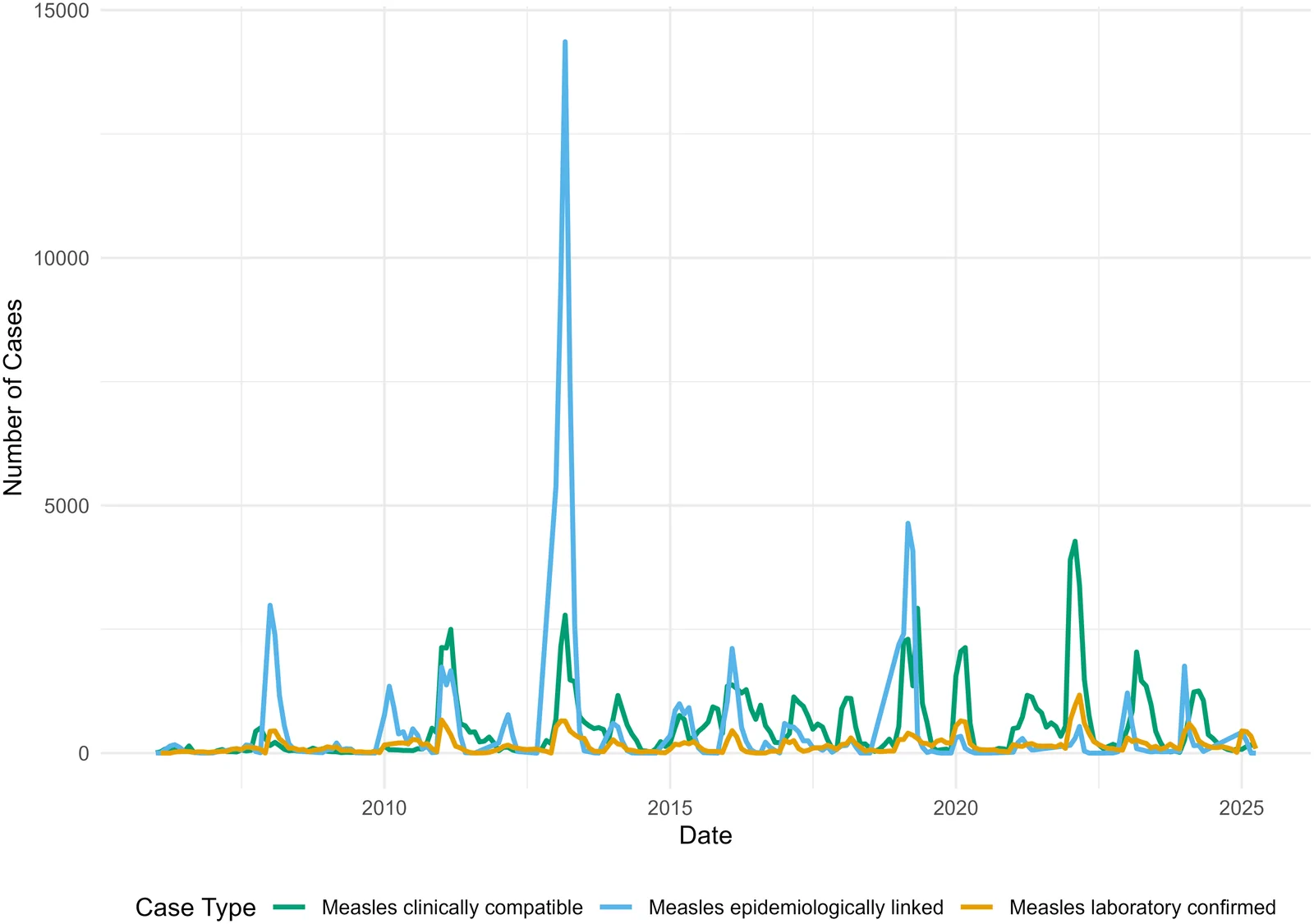
National monthly measles cases in Nigeria, 2006–2025;A time-series line plot showing monthly aggregated measles cases. The figure illustrates strong inter-annual variability and identifies March 2013 as the peak outbreak month (15,010 cases).

**Fig 2:**
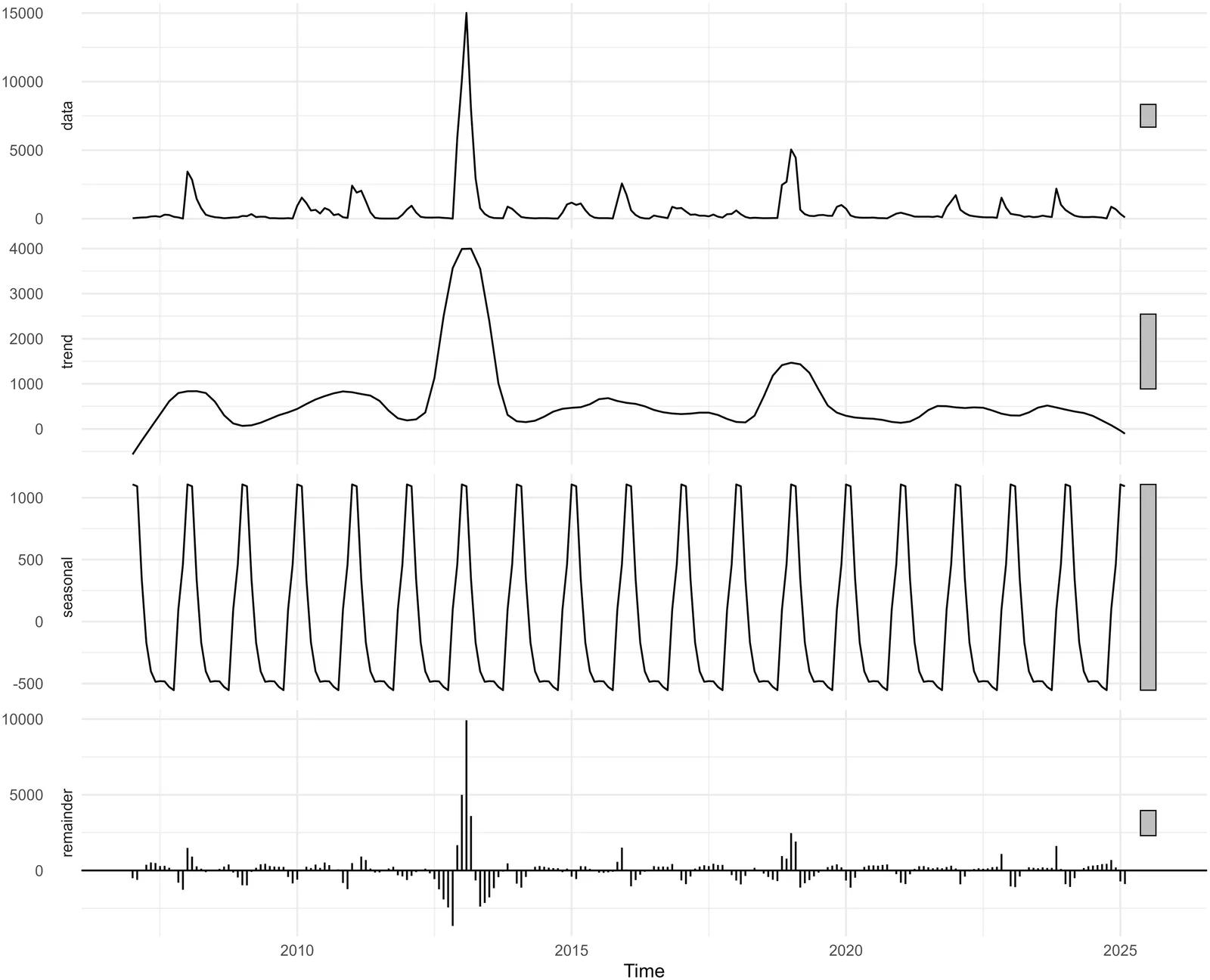
Decomposition of the measles time series into trend, seasonal, and residual components, 2006–2025. The seasonal component shows weak yet consistent peaks from December to February. The trend component highlights fluctuating epidemic cycles with pronounced increases during outbreak years.

Spatial analysis at the state level demonstrated marked geographic heterogeneity. The cumulative burden of measles was heavily concentrated in northern Nigeria. The top five states Katsina, Borno, Kano, Sokoto, and Kebbi collectively reported a substantial proportion of the national case total (Table 2, Fig 3). In contrast, states in the southern regions, such as Cross River and Ebonyi, consistently reported the lowest cumulative cases.

**Table 2:** Top 10 states with the highest cumulative measles burden, 2006–2025. *Ranks states by total case counts, highlighting high-burden northern states.*

State	Total Cases	Mean Monthly Cases	Max Monthly Cases	Years With Cases
Katsina	22,188	101.78	4,073	19
Borno	18,381	84.32	3,574	19
Kano	14,720	67.52	3,609	19
Sokoto	13,773	63.18	1,697	19
Kebbi	12,389	56.83	2,260	19
Jigawa	10,361	47.53	1,317	19
Kaduna	6,196	28.42	1,073	19
Zamfara	4,349	19.95	1,007	19
Yobe	3,958	18.16	483	19
Bauchi	3,522	16.16	919	19

**Fig 3:**
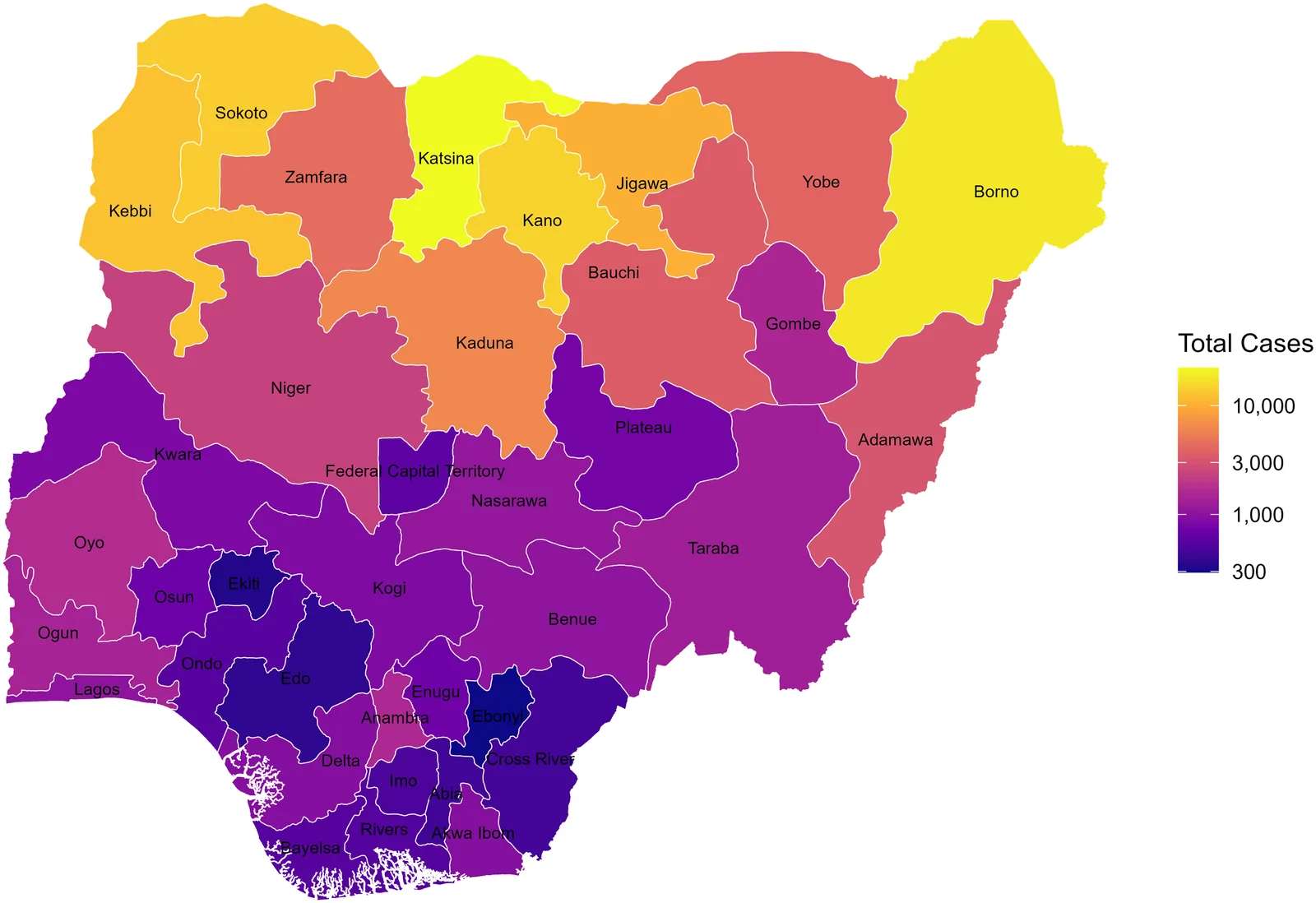
Spatial distribution of cumulative measles cases across Nigeria, 2006–2025; A choropleth map showing state-level total measles burden. Northern states (Katsina, Borno, Kano, Sokoto, Kebbi) appear as high-burden hotspots, while southern/coastal states exhibit low case totals. *Source: Authors*’ *analysis using study data and GADM v4.1 boundary data.*

### 3.3. Identification of spatio-temporal hotspots

Spatial autocorrelation analysis confirmed that the geographic distribution of measles cases was highly clustered. Global Moran’s I analysis showed a statistically significant positive spatial autocorrelation (Moran’s I = 0.397, p < 0.001), indicating that cases were not distributed randomly (Fig 4).

**Fig 4:**
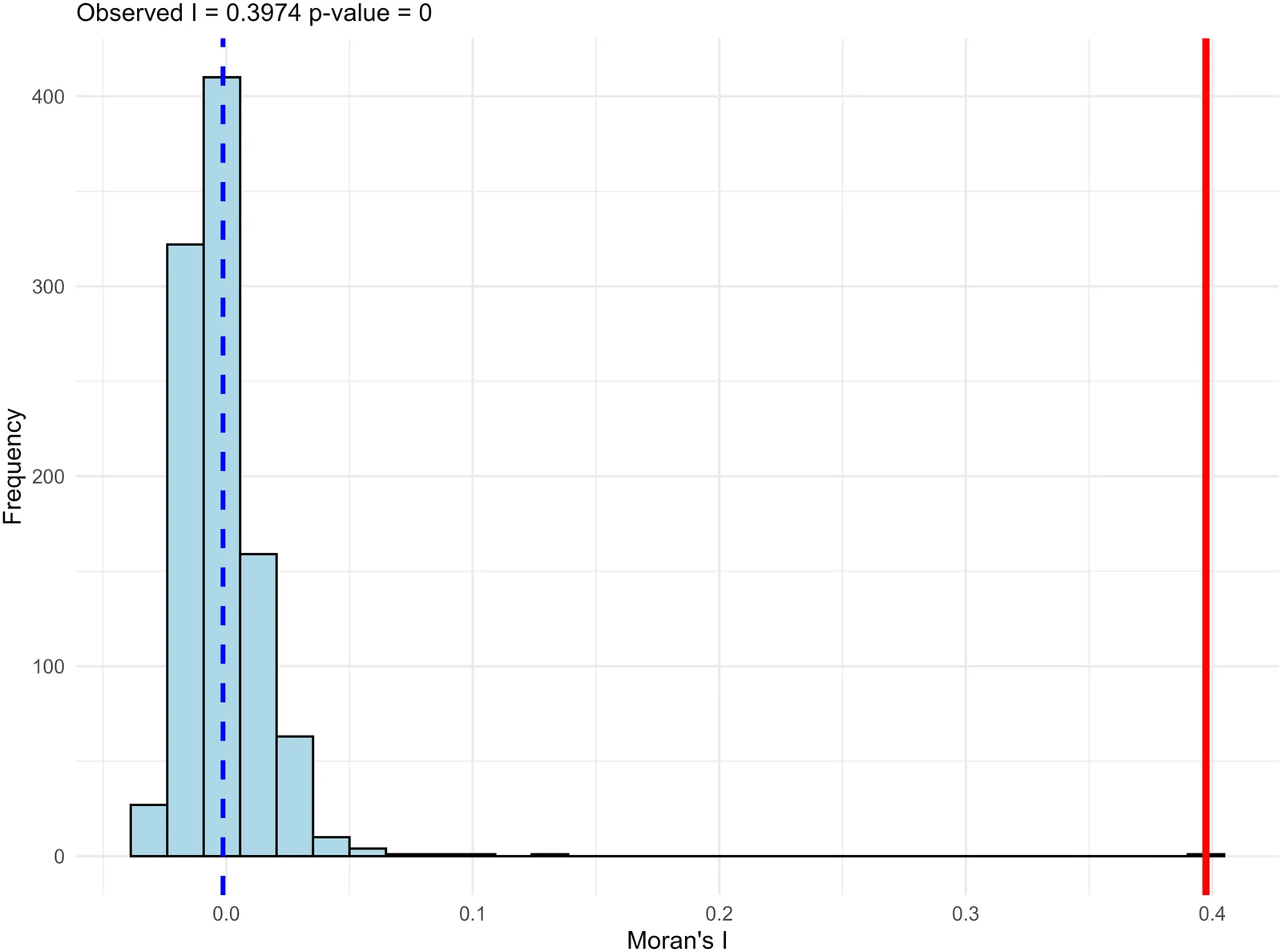
Global Spatial Autocorrelation of Measles Cases in Nigeria (Global Moran’s I);Global Moran’s I analysis demonstrates statistically significant positive spatial autocorrelation in measles incidence across Nigerian LGAs.

Local cluster analysis (LISA) identified 32 LGAs as significant high-high clusters, primarily located in the Northeast and Northwest regions (Fig 5). Similarly, the Getis-Ord Gi* statistic identified 46 LGAs as high-intensity hotspots, forming a contiguous belt across northern states like Borno, Yobe, and Katsina (Fig 6).

**Fig 5:**
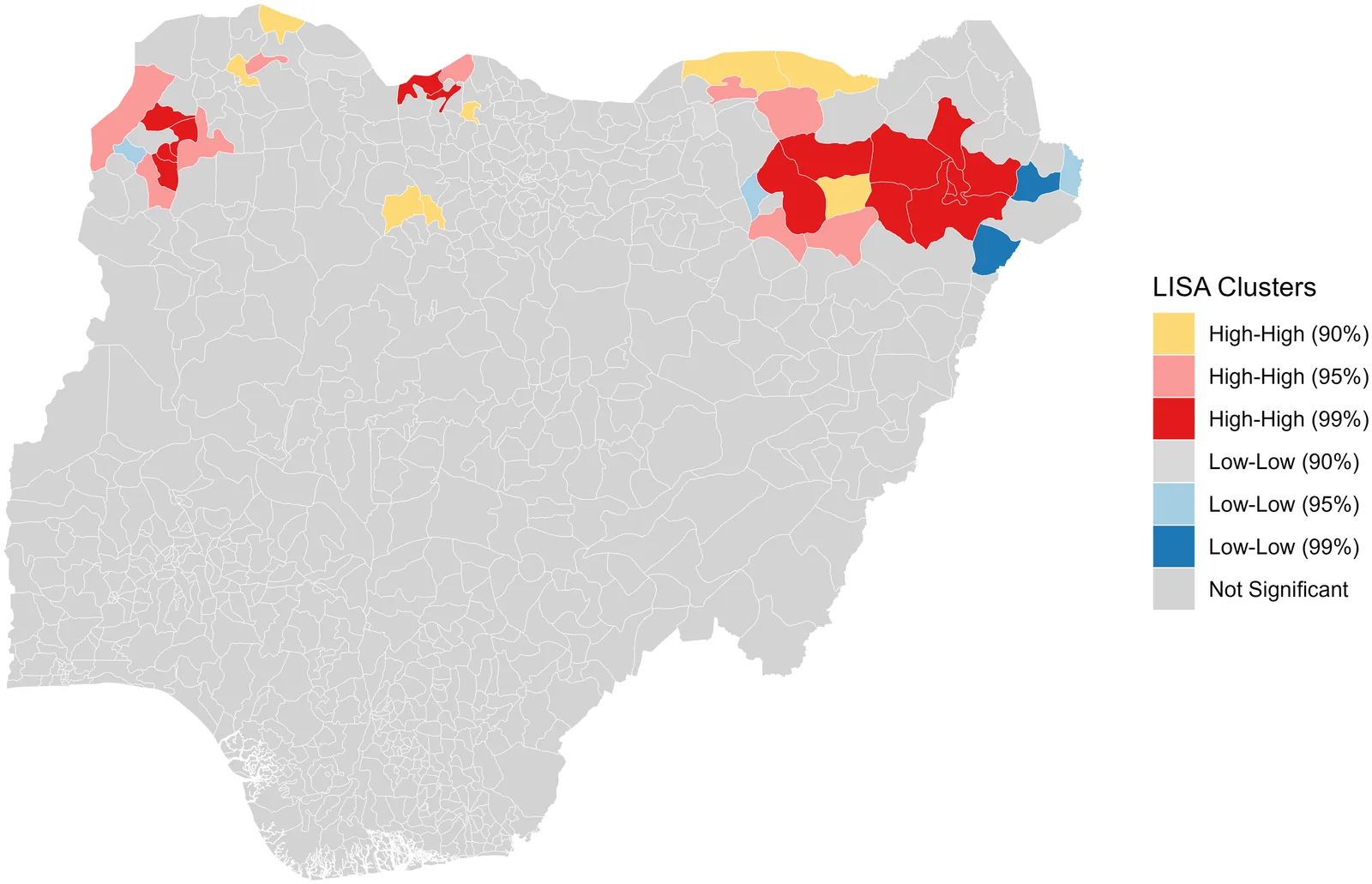
Local Moran’s I (LISA) Clusters of Measles Cases: High-high, low-low, and outlier spatial clusters of measles incidence across Nigerian LGAs at 90%, 95%, and 99% significance levels. *Source: Authors*’ *analysis using study data and GADM v4.1 boundary data.*

**Fig 6:**
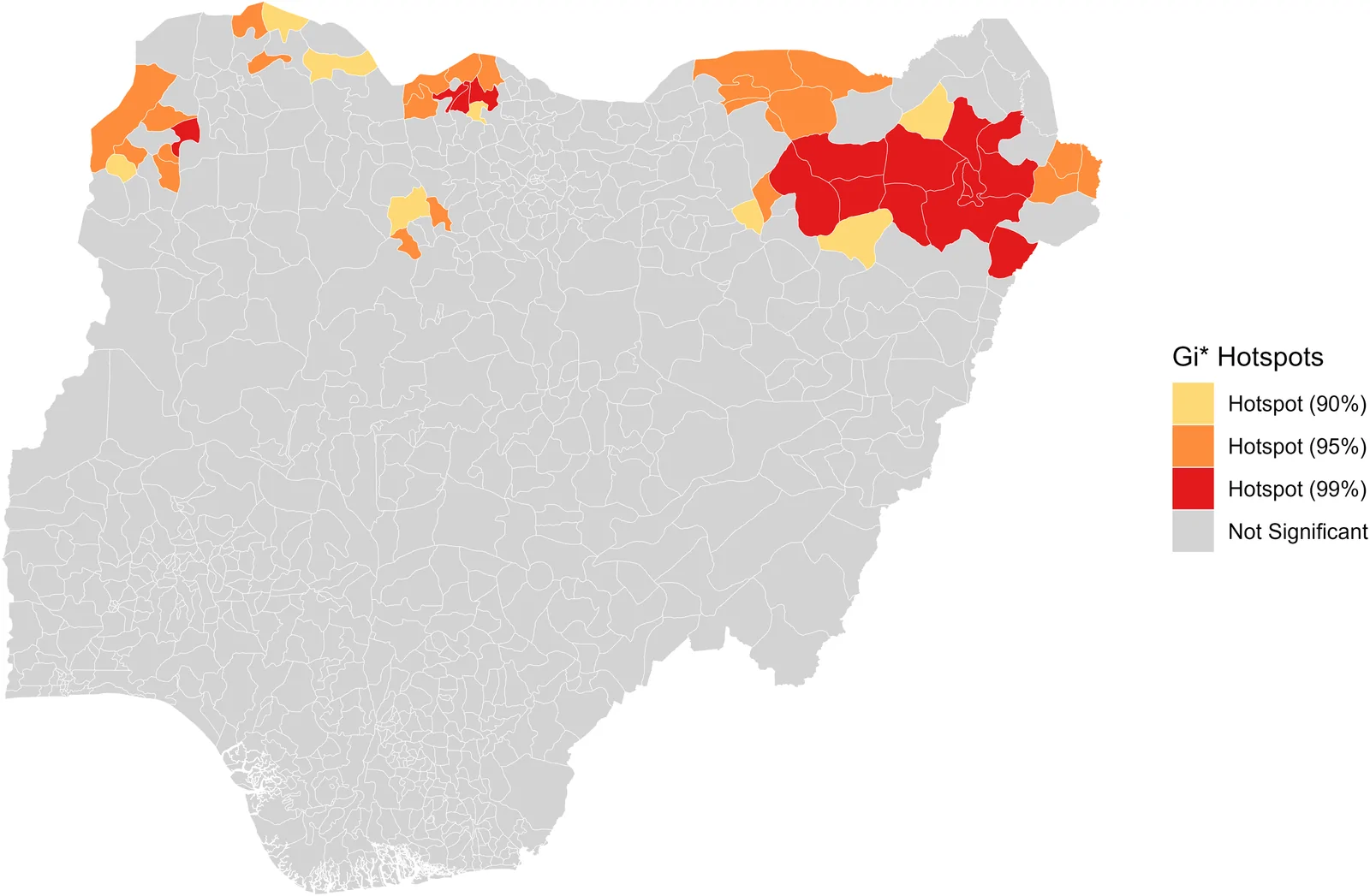
Getis-Ord Gi Hotspot Analysis Results; Statistically significant Gi* hotspots (99%, 95%, 90%) showing concentrations of high measles intensity across northern Nigeria. *Source: Authors*’ *analysis using study data and GADM v4.1 boundary data.*

The temporal dimension of these hotspots was a critical finding. Twenty-five LGAs were high-burden areas in at least 30% of the study years. LGAs such as Damaturu (57.9%), Nguru (52.6%), and Maiduguri (47.4%) demonstrated the highest persistence, indicating entrenched transmission dynamics (Table 3).

**Table 3:** Top 15 most persistent measles hotspot LGAs, 2006–2025.

Rank	LGA	State	Persistence (%)
1	Damaturu	Yobe	57.9
2	Nguru	Yobe	52.6
3	Maiduguri	Borno	47.4
4	Potiskum	Yobe	42.1
5	Bade	Yobe	42.1
6	Jere	Borno	36.8
7	Biu	Borno	36.8
8	Fune	Yobe	36.8
9	Kaga	Borno	31.6
10	Gaidam	Yobe	31.6
11	Kukawa	Borno	31.6
12	Kwaya Kusar	Borno	31.6
13	Gubio	Borno	31.6
14	Gwoza	Borno	31.6
15	Marte	Borno	31.6

### 3.4. LGAs with the highest temporal persistence of measles outbreaks across 19 years

Synthesizing these spatial and temporal findings, an integrated risk classification was developed. This multi-criteria approach categorized a small proportion of LGAs (4.9%, n = 38) as “Persistent High-Risk Hotspots.” Despite their small number, these LGAs accounted for 23.2% of the national case burden, with an average of 1,547 cases per LGA (Table 4). This risk was overwhelmingly concentrated in the north; Of the 38 ‘Persistent High-Risk Hotspots’ identified, 37 (97.4%) were located across Borno, Yobe, Katsina, Kebbi, and Sokoto. Overall, northern LGAs experienced a 6.4-fold higher measles burden than southern LGAs, accounting for 86.4% of all national cases. The final integrated hotspot map clearly delineates these high-risk corridors (Fig 7).

**Table 4:** Distribution of LGAs and measles cases by hotspot priority classification.

Hotspot Category	Number of LGAs	Percentage of LGAs	Total Cases	Average Cases per LGA
Persistent High-Risk Hotspot	38	4.9%	58,783	1,547
High Case Burden	157	20.3%	125,443	799
Low Risk	576	74.3%	65,851	114
Spatial Cluster Only	3	0.5%	3,518	1,173
Total	774	100%	253,595	328

*Summary of LGA classifications based on integrated spatial–temporal measles hotspot analysis. A small number of LGAs (n = 3) were classified as ‘Spatial Cluster Only,’ indicating they were part of a significant spatial cluster but did not rank highly in terms of absolute burden or temporal persistence*

**Fig 7:**
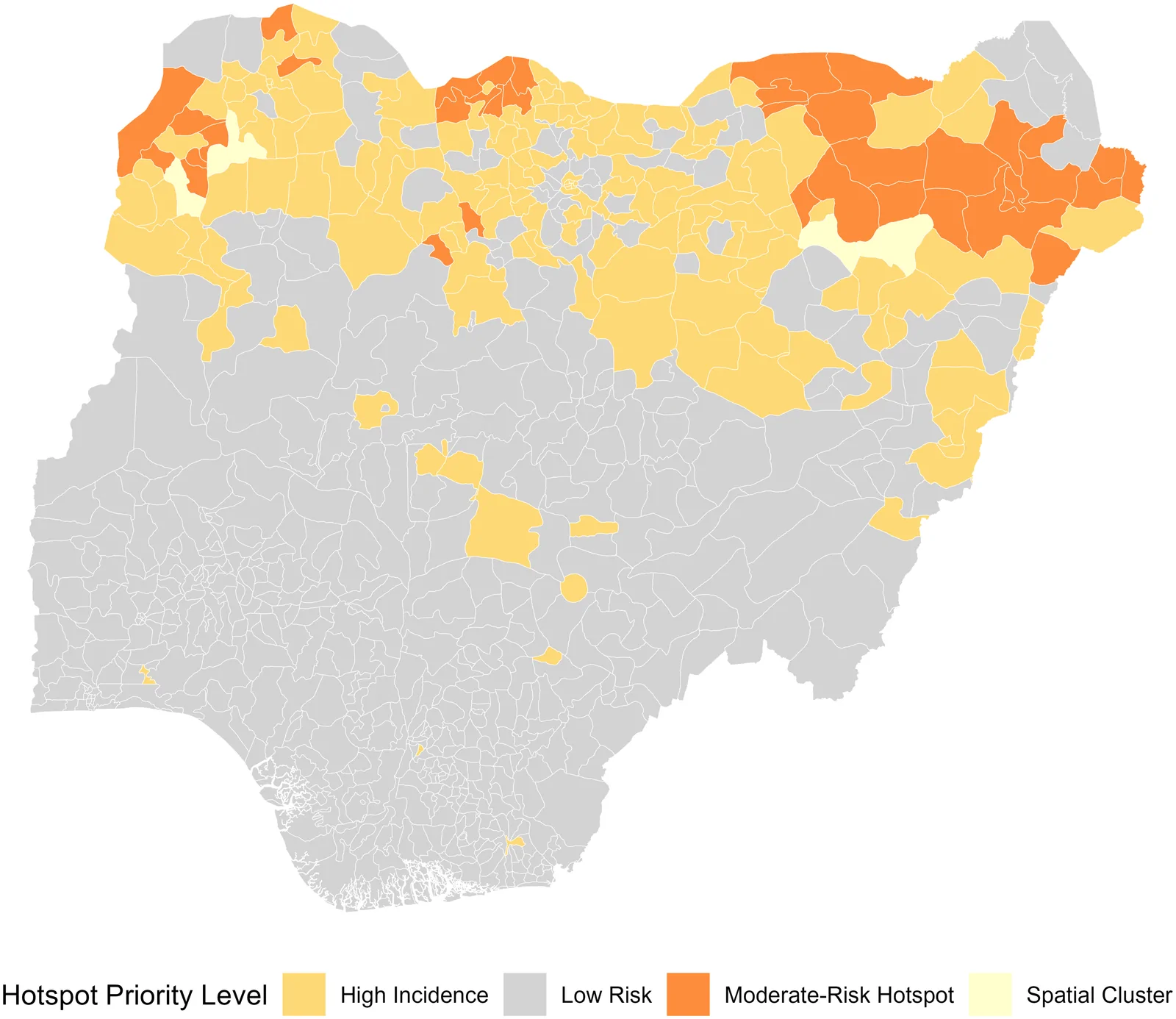
Integrated Measles Hotspot Priority Classification Map (2006–2025): Map showing the four-category integrated hotspot priority classification across LGAs in Nigeria. *Source: Authors*’ *analysis using study data and GADM v4.1 boundary data.*

### 3.5. Vaccination coverage and association with measles case ratio

Analysis of recent vaccination coverage (2021–2024) revealed critical gaps. The national average administrative coverage for the first dose of measles-containing vaccine (MCV1) was 73.8%, while second-dose (MCV2) coverage lagged significantly at 38.4%. Although both showed a steady, improving trend over the period (Fig 8), pronounced geographic disparities persisted. Twenty-four states (64.9%) had MCV1 coverage below 80%, and 34 states (91.9%) had MCV2 coverage below 50% (Fig 9, Table 5).

**Table 5:** State-level vaccination coverage and measles burden (2021–2024).

State	Average MCV1 (%)	Average MCV2 (%)	Total Cases	Case Ratio	Priority Level
Federal Capital Territory	101.6	64.9	125	85.2	Low
Adamawa	97.4	55.3	287	125.4	Low
Nasarawa	95.7	52.1	104	62.8	Low
…	…	…	…	…	…
Bayelsa	40.0	17.8	1,215	1,349.4	Critical
Edo	47.7	23.4	543	289.6	High
Osun	52.6	26.1	327	174.1	High

**Fig 8:**
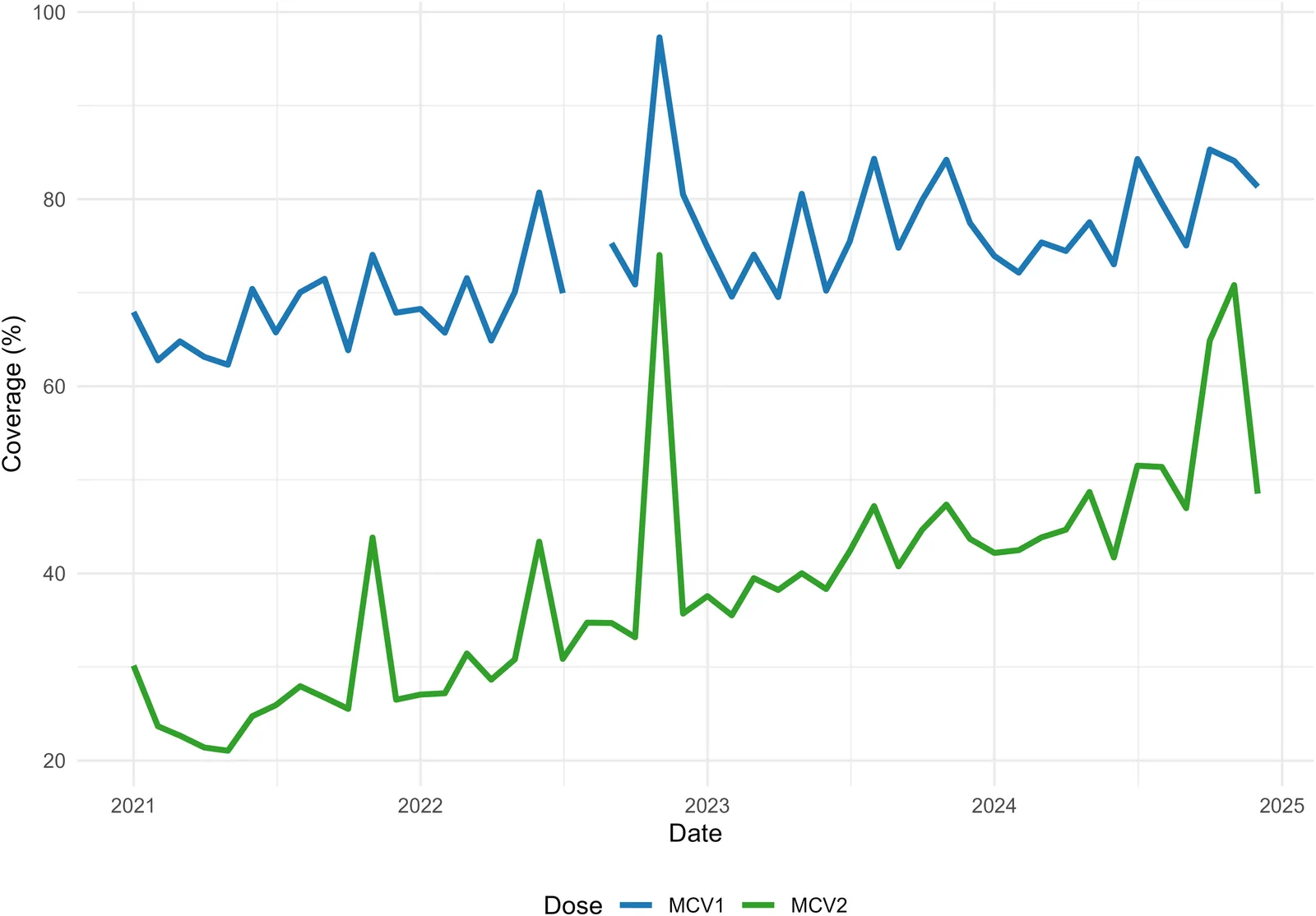
National Trends in MCV1 and MCV2 Vaccination Coverage, 2021–2024; Monthly national averages showing gradual increases in first- and second-dose measles vaccination coverage over the four-year period.

**Fig 9:**
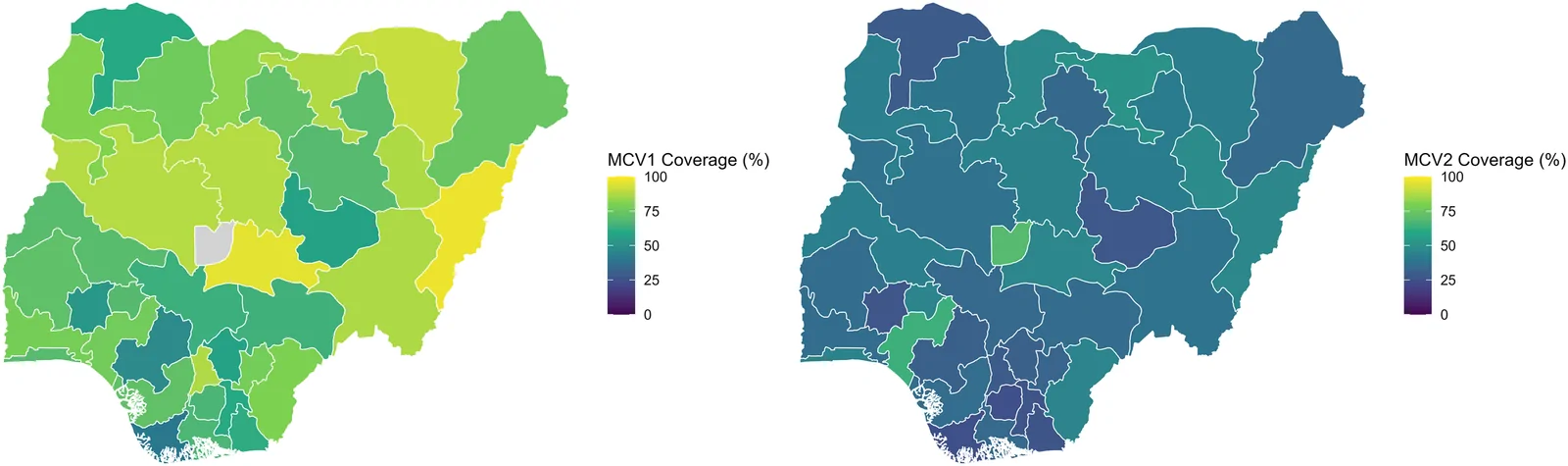
Geographic Distribution of Average MCV1 and MCV2 Coverage Across Nigerian States, 2021–2024; State-level maps illustrating substantial heterogeneity in first- and second-dose measles vaccination coverage. *Source: Authors*’ *analysis using study data and GADM v4.1 boundary data.*

The relationship between vaccination coverage and disease case ratio was complex. A weak, inverse correlation was observed between state-level MCV1 coverage and case ratio (mean r = −0.108). However, the mixed-effects regression model, which accounts for unmeasured, time-invariant state-level characteristics, revealed a significant negative association. In the final adjusted model, higher MCV1 coverage was significantly associated with a reduction in measles case ratio (β = −0.378, SE = 0.090, p < 0.001). The association for MCV2 coverage was not statistically significant, and no interaction was found between the two doses (Fig 10). Model diagnostics confirmed the assumptions of residual independence (Durbin-Watson = 1.965) and absence of influential observations (maximum Cook’s distance = 0.071, below the 0.5 threshold). However, the Shapiro-Wilk test indicated some deviation from normality (p < 0.001), which is expected given the log-transformation of the outcome variable. The maximum Variance Inflation Factor (VIF) was 9.78, indicating acceptable levels of collinearity among predictors. The models indicated that most variability in case ratio was attributable to stable differences between states (conditional R² = 0.120), rather than being explained by the predictors over time (marginal R² = 0.022). It is important to note that the marginal R² for the models was low (~0.02), indicating that the predictors (MCV1, MCV2, Time) explained a small proportion of the variance in case ratio over time. The higher conditional R² (~0.12) suggests that a larger portion of the explainable variation is attributable to stable, unmeasured differences between states

**Fig 10:**
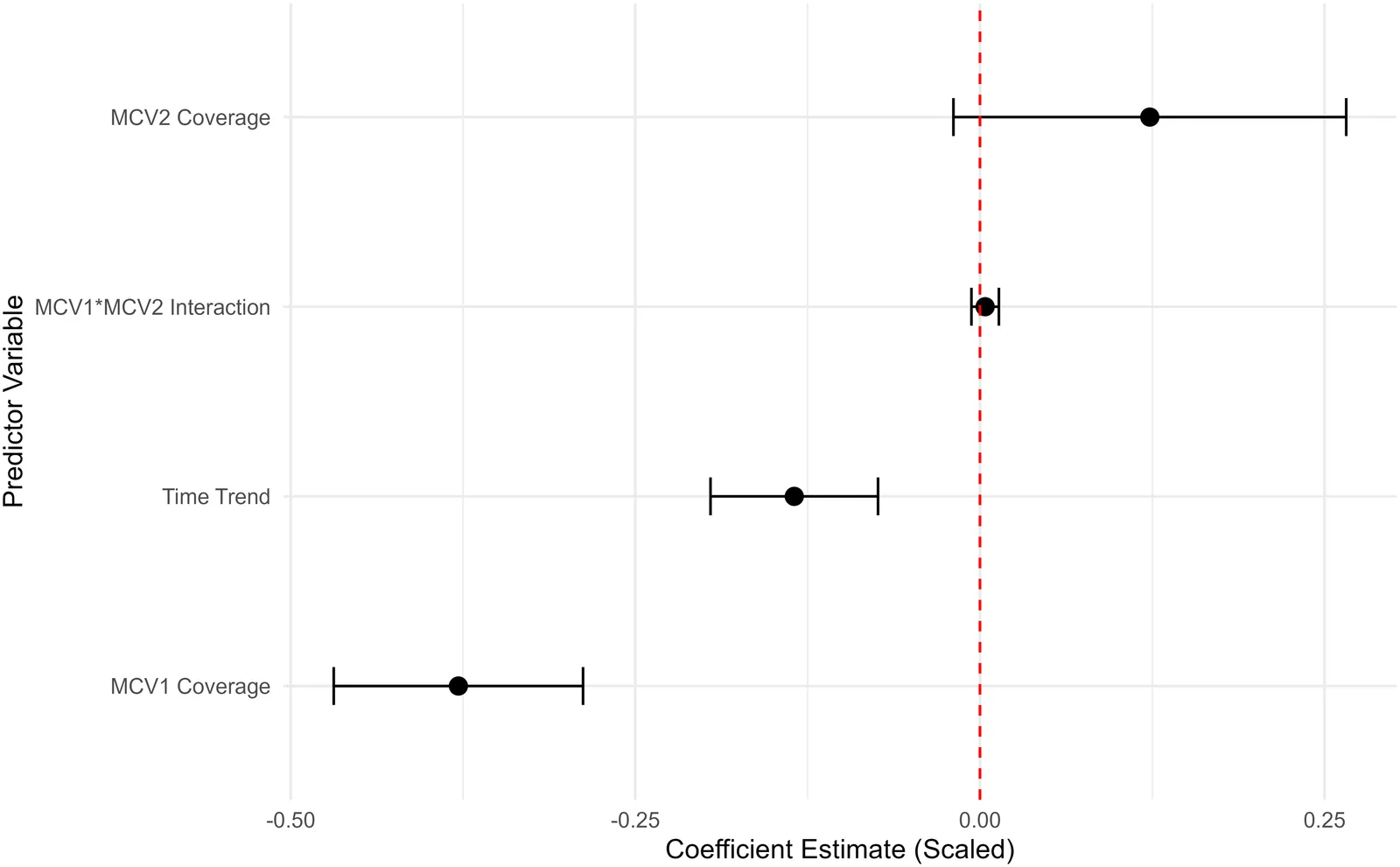
Fixed-Effect Coefficients from the Mixed-Effects Model (Model 3);Coefficient estimates with 95% confidence intervals showing the relative contribution of each predictor to measles Case Ratio.

The ROC analysis confirmed that MCV1 coverage has poor discriminative ability for identifying outbreak months (AUC = 0.519). The low sensitivity (46.9%) indicates that relying on a 75.1% coverage threshold would miss more than half of outbreak months, while the modest specificity (59.4%) means that nearly 40% of non-outbreak months would be incorrectly flagged as high-risk. The particularly low PPV (27.9%) suggests that using this threshold for early warning would generate many false alarms, potentially diverting limited resources from genuine outbreaks. These findings underscore the limitations of using state-level administrative coverage alone for outbreak prediction and reinforce the need for more granular, sub-state surveillance data and additional predictive indicators [28,29]. Outbreak risk was non-linear, unexpectedly peaking in the 85–95% coverage group. Based on the synthesis of low MCV1 coverage and high case ratio, a state-level intervention priority classification was established. Six States: Bayelsa, Enugu, Plateau, Akwa Ibom, Ekiti, and Kwara were classified as Critical Priority. An additional ten states were categorized as High Priority, representing key targets for intensified immunization activities to mitigate future outbreaks (Fig 11).

**Fig 11:**
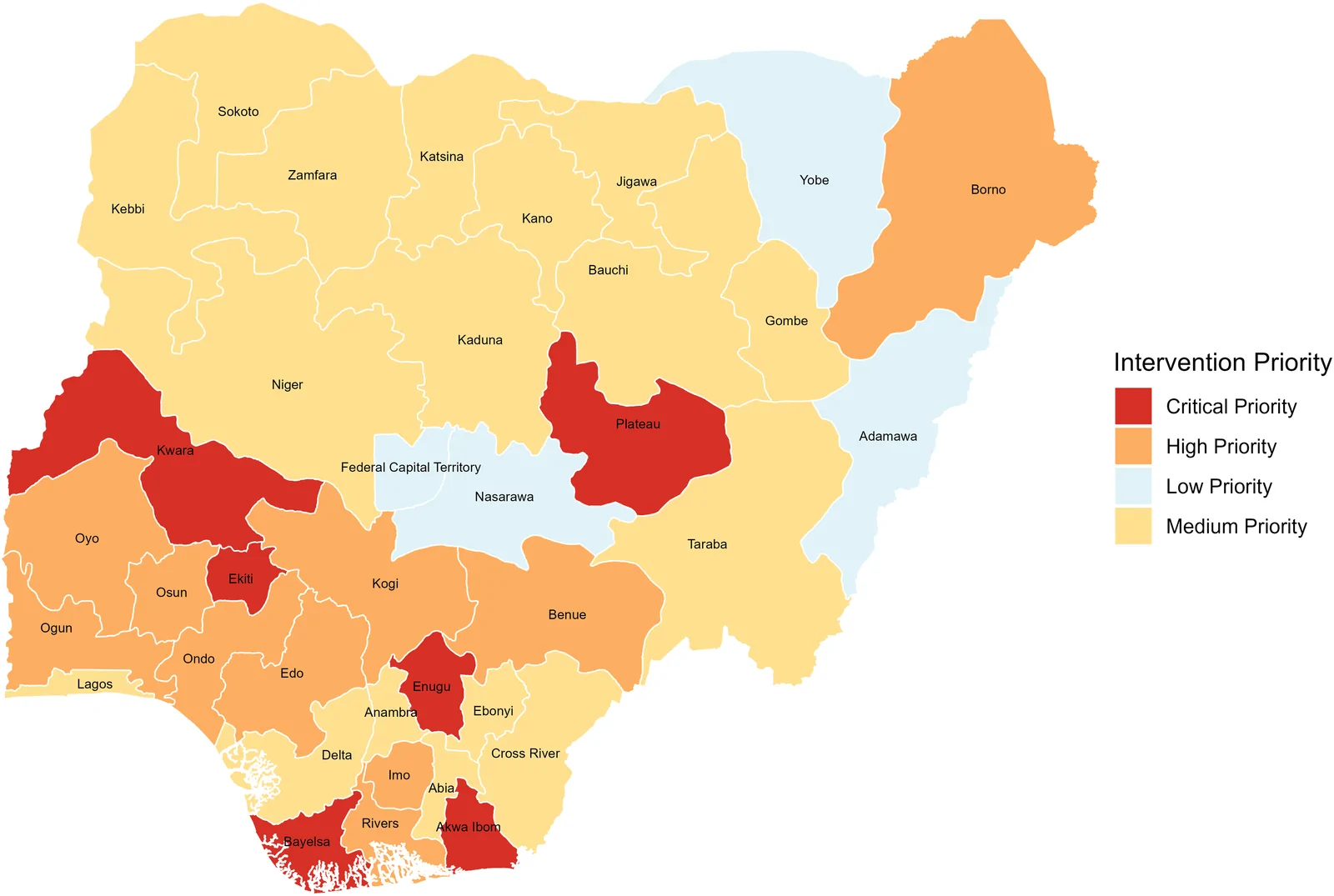
State-Level Prioritization for Measles Vaccination Interventions; Priority categories derived from combined MCV1 coverage and measles case ratio. metrics to support targeted immunization strategies. Source: Authors’ analysis using study data and GADM v4.1 boundary data.

## 4. Discussion

This is a nationwide spatio-temporal study that gives a thorough evaluation of 19-year measles epidemiology in Nigeria. Our results indicate a pattern that is predictable and, nevertheless, alarming: measles has continued to impact disproportionately unvaccinated young children in northern Nigeria, with the spread of the virus rooted in certain and persistent hotspots. The low correlation between the coverage of routine vaccination and disease burden at the population level highlights that the existing immunization measures are not adequate to break the transmission, and a paradigm shift toward geographically and demographically targeted interventions is needed.

### 4.1. The unchanged face of measles: Vulnerable populations and surveillance gaps

Descriptive epidemiology of measles has been quite consistent over the years in Nigeria highlighting the perennial failure to target the most vulnerable. The concentration of cases (61.6%) among children aged 1–4 years is a classic hallmark of measles in populations with sub-optimal vaccination coverage, where the virus rapidly finds susceptible individuals as maternal immunity wanes [22,23]. This trend is reported consistently throughout sub-Saharan Africa and in neighboring countries such as the Democratic Republic of Congo [24]. The stark finding that 68.9% of cases occurred in individuals with no history of vaccination points directly to a large and enduring pool of zero-dose children, which is the primary driver of measles outbreaks globally [13,25].

In addition, the case classification information, whereby only 14.4% of the cases were lab confirmed, tells of paramount flaws in the surveillance system. Although this over-reliance on clinical and epidemiological categorization is typical of resource-limited environments, it may cause misdiagnosis and imprecise representation of circulating pathogens, with diseases such as rubella potentially sharing similar symptoms [26]. This observation is supported by WHO reviews of measles surveillance in the African region that frequently report few rates of case investigation and laboratory confirmation [16]. Furthermore, Umar et al.[27] argue that reducing measles persistence in settings like Nigeria requires a comprehensive approach that extends beyond improving MCV1 and MCV2 coverage. It necessitates robust post-vaccination surveillance, diligent vaccine safety monitoring and adverse event reporting, and sustained strategies to maintain public confidence in immunization programs.

### 4.2. Entrenched hotspots and the limits of spatial analysis

The analysis of geographical clustering of measles cases showed significant clustering, which was consistent over space and time. The existence of high-high clusters and Gi hotspots that have created a continuous belt in the Northeast and Northwest- especially in Borno, Yobe and Katsina- has over a decade shown the same results as the recent geospatial findings [28,29]. LGAs like Damaturu and Maiduguri are still perennial hotspots, implying an endemic transmission and not occasional outbreak. Similar persistence is observed in conflict-affected regions of Afghanistan and Pakistan, where insecurity disrupts immunization services [6].

The 6.4-fold higher burden in the north compared to the south underscores entrenched socio-economic and health system inequities Among the drivers are poverty, low female literacy, large families, and insecurity, which are reported in previous studies in Nigeria [16,30,31]. These patterns have been combined into a useful prioritization tool as our integrated risk map, but this tool must be coupled with spatial knowledge of these barriers along with local knowledge. Alongside these immunization efforts, effective risk communication and strategies to counter misinformation are crucial. As Abdaljaleel et al.[32] highlight, building and maintaining public trust is a critical component of addressing persistent vaccination gaps and preventing future outbreaks, particularly in contexts where immunization programs face significant challenges.

### 4.3. The complex role of vaccination in a heterogeneous landscape

The interaction between immunization coverage and measles case ratio in Nigeria is multifaceted and cannot be approached through a national-level approach only. Our finding of a statistically significant negative association for MCV1 (β = −0.378, p < 0.001) is consistent with the literature; however, the low marginal R² (0.022) indicates that MCV1 coverage explains only a small proportion of the variance over time. This underscores the importance of distinguishing between statistical significance, effect size, and predictive performance, as has been observed in similar studies in India and Ethiopia, where the association between administrative coverage and disease case ratio was also weak due to data quality gaps, the overriding influence of high birth rates, and the overwhelming influence of SIAs [33,34]. Nevertheless, MCV1 showed a statistically significant negative association in the mixed model, which highlights the point that vaccination is associated with reduced disease burden at the population level, but the effect is obscured by deep heterogeneities on the state level.

The model’s conditional R² suggests that most of the explainable variation is due to fixed differences between states, such as historical health system performance, infrastructure, and sociocultural factors, which are not captured by monthly coverage Because of the reported constraints of routine immunization (RI), SIAs continue to be the foundation of the way measles is controlled, and its influence is intimately connected with the spatial patterns which we have found. The presence of heavy concentration of spatio-temporal hotspots in the northeast and the northwest is a result of the interaction between the chronic low coverage of RI, the high fertility, and the irregular SIA reach and quality. There is evidence that SIAs can be used to address immunity gaps, and a multicountry analysis has identified that they cover an estimated 2/3 of zero-dose children that are not covered by routine services [35]. This is consistent with our data, where the northern states, despite lower RI, rely heavily on SIAs for population immunity, whereas southern states benefit from more consistent routine services. In Nigeria, in particular, SIAs were found to supplement RI by inoculating those children who have never been inoculated in the regular pathways (Omoleke et al., 2020) [36]. This creates a fundamental difference in the nature of herd immunity between the regions, explaining why our national-level model had limited explanatory power.

The non-significant association observed for MCV2 was anticipated considering that it is newly introduced and has low coverage in the country (38.4%). This finding may mask important subnational variations. Recent data indicates that the north gained more MCV2 coverage than the south since its introduction in 2019 but starting at a far lower level [11]. This suggests that Nigeria is currently in a crucial transition phase in which the population-level association of MCV2 coverage with reported case ratios may not yet be detectable.

The ROC analysis confirmed that MCV1 coverage is a very weak predictor of outbreak months (AUC = 0.519), a finding consistent with the low explanatory power of the mixed-effects model (marginal R² = 0.022). This suggests that the association between vaccination coverage and reported cases is obscured by many other factors, and any apparent relationship should be interpreted with caution. The paradoxical finding that outbreak risk appeared highest in states with 85–95% MCV1 coverage should be interpreted in this context. We propose three non-mutually exclusive explanations. First, there is a high risk of surveillance and reporting bias: states with better health systems have higher reported vaccination coverage and more sensitive case detection and reporting, which results in an artificial relationship between high vaccination coverage and high reported incidence. Second, the phenomenon of case importation and focal outbreaks may be at play. Urban centers or states with relatively good overall coverage can act as hubs that receive infected individuals from surrounding low-coverage areas; outbreaks can then ignite within dense, localized pockets of susceptible individuals (e.g., in specific slums or hard-to-reach communities) that are not reflected in the state-level average. Third, reactive vaccination in confirmed outbreaks may temporarily artificially boost the coverage numbers in the same regions with high transmission, which makes coverage an effect and not a cause of the outbreak. This paradox adds to the inability to predict risks by using only state-level administrative coverage and makes it important to have sub-state, granular data on immunity gaps.

### 4.4. Implications for a tailored elimination strategy

The clear north-south divide demands two distinct strategic approaches:

- Northern Nigeria (High‑Burden, SIA‑Dependent): Although RI strengthening constitutes a long-term objective, the immediate effect resides in maximizing SIAs this involves frequency and including more age groups within the high-risk LGAs such as 5–14 years of age. This strategy, which has been effectively proposed in high-transmission areas, deals with accrued vulnerability in older children [37].
- Southern Nigeria (Lower‑Burden, Routine‑Plus): The focus must shift to fortifying the routine program states like Bayelsa and Ekiti, classified as “Critical Priority” due to low RI coverage, require intensive routine immunization efforts. Subsequently, less frequent, targeted SIAs (“mop‑ups”) can cover remaining susceptible pockets an approach supported by evidence from transitioning regions toward elimination [38].
- MCV2 Regional Gains: Although national coverage remains modest (38.4%), northern zones have achieved larger increases, reaching 35–42% MCV2, whereas southern zones lag at 22–31%, highlighting a critical transitional phase before full population-level impact [11].

### 4.5. Limitations

This study has some limitations. First, the ecological design precludes causal inference; associations between vaccination coverage and case ratios should not be interpreted as causal effects. Second, the use of live births as the denominator for case ratios, while necessary due to data constraints, does not account for population size beyond the birth cohort, and states with different age structures may have case ratios that are not directly comparable. Third, only 14.4% of cases were laboratory-confirmed; the remainder were clinically compatible or epidemiologically linked, potentially introducing misclassification bias. However, a sensitivity analysis using only laboratory-confirmed cases showed similar spatial hotspot patterns (results not shown). Fourth, administrative vaccination coverage data may be subject to reporting inaccuracies and overestimation. Fifth, the mixed-effects model explained only a small proportion of the variance over time (marginal R² ≈ 0.02), indicating that unmeasured factors including health system performance, sociocultural barriers, and supplemental immunization activities are major drivers of measles transmission not captured by routine coverage metrics. Finally, our analysis of vaccination effects was restricted to 2021–2024, limiting generalizability to earlier periods when coverage patterns differed. Despite these limitations, the long-term surveillance dataset and multi-method approach provide robust evidence for understanding measles transmission dynamics in Nigeria.

## 5. Conclusion

In conclusion, despite decades of control efforts, measles transmission in Nigeria remains starkly divided between northern and southern regions, which require distinct strategic responses. Progress towards elimination requires confronting this reality with precision public health. In the north, this means acknowledging the limitations of RI and deploying more aggressive, frequent, and broader SIA campaigns to reduce endemic transmission in persistent hotspots. In the south, the goal is to defend gains by fortifying routine systems and moving towards a quality-focused, surveillance-driven response. Sustainable progress hinges on this differentiated, data-driven approach that addresses the unique epidemiological realities of each region.
