# Supplementary material for: Spatio-temporal dynamics, transmission hotspots, and vaccination gaps driving measles persistence in Nigeria, 2006–2025
Source: PLoS One. 2026 Oct 27;21(10):e0359837. doi: 10.1371/journal.pone.0359837 (PMC13712523; doi:10.1371/journal.pone.0359837)
Supplement: Supplementary file 1 [file pone.0359837.s002.pdf]

Manuscript Title: Spatio-Temporal Dynamics, Transmission Hotspots, and Vaccination Gaps Driving Measles Persistence in Nigeria, 2006–2025

Responses to Editor Comments

Editor Comment	Response	Location in Manuscript
**1. PLOS ONE Style Requirements** - Please ensure manuscript meets PLOS ONE's style requirements, including file naming.	We have carefully reviewed the PLOS ONE style templates and revised the manuscript accordingly. We have ensured that all formatting, headings, references, and file naming conventions follow the journal's requirements. All supporting information files have been appropriately named and formatted.	Throughout manuscript
**2. Code Sharing Guidelines** - Please ensure all author-generated code is made available without restrictions upon publication.	We confirm that all R code used for data analysis, including time-series decomposition, spatial autocorrelation (Local Moran's I and Getis-Ord Gi*), and linear mixed-effects modeling, will be made publicly available without restrictions upon publication. The complete code and generated datasets (excluding restricted surveillance data) have been deposited in **Zenodo** and will be accessible immediately upon publication. The DOI will be provided in the Data Availability Statement.	Data Availability Statement (submission form)
**3. Data Availability Statement -** Please revise the data availability statement to specify how data will be made freely accessible upon acceptance.	We have revised the data availability statement to clearly outline the data sharing plan: (1) All R codes and generated analytical datasets (e.g., aggregated LGA-level case ratios, vaccination coverage metrics, spatial weights matrices) will be made freely available on **Zenodo** without restrictions. (2) The raw surveillance data used in this study are owned by the Nigeria Centre for Disease Control (NCDC) and the WHO Regional Office for Africa (WHO AFRO). Access to these data is governed by institutional data sharing policies and ethical approvals. These data are available upon reasonable request to the NCDC (data@ncdc.gov.ng), subject to institutional permission and appropriate data use agreements. This approach ensures transparency and	Data Availability Statement (submission form)

	reproducibility while respecting the data governance framework of the original data custodians.	
**4. Ethics Statement Location** - Ethics statement should only appear in the Methods section.	We have confirmed that the ethics statement appears only in the Methods section (Section 2.4) and has been removed from any other section of the manuscript.	Section 2.4 (Methods)
**5. Copyrighted Figures (Figures 3, 5, 6, 7, 9, 12)** - Please address copyright concerns for map/satellite images.	We confirm that ALL figures (including Figures 3, 5, 6, 7, 9, and 12) were created by the authors using our own data, publicly available shapefiles (GADM version 4.1, which is freely available for academic use), and original R code. These figures do NOT contain any proprietary Google Maps, Google Earth, or any third-party copyrighted content. The base shapefiles were sourced from the public domain Database of Global Administrative Areas (GADM). We have updated all figure captions to clearly state the source of the spatial data and confirm that the maps are original creations. Since all figures are our own work, no permissions are required, and they can be published under CC BY 4.0.	Figure captions (Figures 3, 5, 6, 7, 9, 12)
**6. Supporting Information Captions** - Please include captions for Supporting Information files at the end of the manuscript.	Supporting Information captions have been added at the end of the manuscript, before the References section. All in-text citations to supporting information have been updated to match accordingly.	End of manuscript (before References)
**7. Reviewer-Recommended Citations** - Please review and evaluate publications recommended by reviewers.	We have carefully reviewed all recommended citations. We have incorporated citations to Abdaljaleel et al. (2024) [ref 32] in the Discussion section regarding risk communication and misinformation control, and Umar et al. (2025) [ref 27] regarding post-vaccination surveillance and vaccine safety monitoring. These references have been critically evaluated and are relevant to our work.	Discussion section (references 27 and 32)

Responses to Reviewer #1

Reviewer Comment	Author Response	Location in Manuscript
**1. Abstract Period Inconsistency** - Abstract states "focused 4-year analysis of vaccination records" but does not specify years (2021-2024), while overall study period cited as 2006-2025. Please explicitly mention the vaccination analysis period in the Abstract.	We agree with this important observation. The Abstract has been revised to explicitly specify the vaccination analysis period as 2021–2024 in both the Objective and Methods sections. The overall study period (2006–2025) remains clearly stated for the spatio-temporal trend analysis.	Abstract (Objective and Methods sections)
**2. Zero-Dose Definition** - Please correct the contradictory phrase "zero-dose children who received at least one dose" in the Introduction. Zero-dose means no doses received.	We thank the reviewer for identifying this critical error. The phrase has been corrected to accurately describe zero-dose children. The revised text now reads: "Nigeria represented about 47 percent of the total number of zero-dose children (those who have not received any routine vaccinations) in the world in 2022."	Introduction (paragraph 2)
**3. Figure 3 Caption** - Caption states "2007-2025" while main text consistently refers to 2006-2025.	We have corrected the caption of Figure 3 to read "2006–2025" to ensure consistency with the main text and the rest of the manuscript.	Figure 3 caption
**4. Data Inconsistency** - Text states average cases per LGA as 1,558 cases, while Table 4 shows 1,547 cases. Please reconcile these numbers.	We thank the reviewer for catching this discrepancy. Upon verification, we confirmed that the correct average is 1,547 cases per LGA. We have updated the text in the Results section to reflect 1,547 cases (previously stated as 1,558) to match Table 4.	Results section (paragraph 7)
**5. Missing Figure 10** - Figure 9 is followed directly by Figure 11, with no Figure 10 appearing.	We thank the reviewer for catching this figure numbering error. The manuscript has been corrected to ensure sequential figure numbering. The figure previously numbered as Figure 11 has been renumbered to Figure 10, and the subsequent figure (previously Figure 12) has been renumbered to Figure 11. All in-text citations have been updated accordingly.	Throughout manuscript (Figures 10 and 11)

**6. Citation Recommendation** - Consider citing Abdaljaleel et al. (2024) to support risk communication and misinformation-control strategies alongside targeted measles immunization efforts.	We have carefully reviewed the recommended citation and agree that it is highly relevant to our discussion of addressing persistent vaccination gaps and public trust. We have incorporated this citation in the Discussion section, specifically in the paragraph discussing the need for comprehensive approaches beyond vaccination coverage.	Discussion section (paragraph 5) [Reference 32]
**7. Duplicate Sentence** - In the Discussion, within the paragraph discussing paradoxical results, there is a clear duplication: "Second, there is the option of the phenomenon of case importation and focal outbreaks." immediately followed by "Second, the phenomenon of case importation and focal outbreaks may be at play."	We have removed the duplicate sentence. The revised text now reads: "Second, the phenomenon of case importation and focal outbreaks may be at play. Urban centers or states with relatively good overall coverage can act as hubs that receive infected individuals from surrounding low-coverage areas..."	Discussion section (paragraph 7)
**8. Citation Recommendation** - Consider citing Umar et al. (2025) in the Discussion, particularly under "The Unchanged Face of Measles" subsection or the paragraph discussing the need for geographically and demographically targeted interventions.	We have reviewed and incorporated this citation in the Discussion section, within the subsection "The Unchanged Face of Measles: Vulnerable Populations and Surveillance Gaps." The citation supports the argument that reducing measles persistence requires not only improved MCV1/MCV2 coverage but also robust post-vaccination surveillance, vaccine safety monitoring, AEFI reporting, and strategies to sustain public confidence.	Discussion section (paragraph 2) [Reference 27]
**9. Study Limitations** - Limitations are currently scattered throughout the Discussion. Please consolidate them into either a separate "Limitations" sub-section or a single paragraph at the end of the Discussion, just before the Conclusion.	We agree with this suggestion and have consolidated all study limitations into a dedicated "Limitations" subsection at the end of the Discussion, immediately before the Conclusion. This includes limitations regarding ecological design, live births denominator, laboratory confirmation rates, administrative coverage data quality, model explanatory power, and generalizability.	Discussion section (Limitations subsection)

Responses to Reviewer #2

Reviewer Comment	Author Response	Location in Manuscript
**1. Mixed-Effects Model - Temporal Autocorrelation** - Please check temporal autocorrelation among repeated monthly observations and include more details regarding model diagnostics (residual assumptions, heteroscedasticity, influential observations, collinearity between MCV1 and MCV2).	We thank the reviewer for this important methodological suggestion. We have now conducted comprehensive diagnostic assessments of the mixed-effects models, which are detailed in Section 2.3.2. Specifically: Durbin-Watson test for temporal autocorrelation (result: 1.965, indicating no significant autocorrelation), Shapiro-Wilk test for normality (noted deviation as expected with log-transformation), Variance Inflation Factor (VIF = 9.78, below 10, indicating acceptable collinearity), and Cook's distance (max = 0.071, below 0.5, indicating no influential observations). We have added these details to the manuscript.	Section 2.3.2 (Methods)
**2. Causal Language** - Please exercise greater care when using causal language; expressions such as "protective effect" or "strong protective impact" imply causal relationships when the analysis shows associations.	We agree with the reviewer's important distinction. We have systematically revised the manuscript to use more cautious language throughout. "Protective effect" has been replaced with "negative association" or "statistically significant association." "Strong protective impact" has been revised to "statistically significant association." The Abstract, Results, Discussion, and Conclusion have all been revised to reflect associational rather than causal language. We have also added explicit acknowledgment of the ecological design limitation precluding causal inference.	Abstract, Results, Discussion, Conclusion throughout
**3. MCV1 Threshold and ROC Analysis** - AUC = 0.519 shows weak discriminatory power; the threshold should not be regarded as a strong or significant protective threshold. Please provide more	We have significantly expanded the ROC analysis section and revised our interpretation. The AUC of 0.519 confirms very poor discriminative ability. We now provide detailed information on sensitivity (46.9%), specificity (59.4%), positive predictive value	Section 2.3.3 and Results section (paragraph on ROC analysis)

details on ROC analysis including sensitivity, specificity, and confidence intervals.	(27.9%), and confidence intervals for the AUC. We explicitly state that MCV1 coverage is a very weak predictor of outbreak months and that the threshold should not be considered a "protective threshold" in any meaningful sense.	
**4. Use of Live Births as Denominator** - Please always refer to this result as a "case ratio" and not use terms "incidence" or "transmission risk" interchangeably. Make clearer the limitations.	We have carefully revised the manuscript to consistently use the term "case ratio" (per 100,000 live births) throughout and have avoided using "incidence" or "transmission risk" interchangeably. We have also expanded the discussion of limitations in the dedicated Limitations subsection, specifically addressing that live births denominator does not account for population size beyond the birth cohort and may limit comparability across states with different age structures.	Throughout manuscript; Limitations subsection
**5. Spatial Analysis - Justification for 6-Nearest Neighbors** - Please give clearer justification for choosing six neighbors and explain if other spatial weight structures were considered.	We have added a detailed justification for the 6-nearest-neighbors approach in the Methods section (2.2.3). We explain that this choice was guided by the need to capture local transmission extending beyond immediate administrative boundaries, approximating the estimated mean radius of measles importation and secondary spread. We also clarify that alternative weights (queen contiguity and fixed-distance bands) were explored, but the 6-nearest-neighbors matrix provided the most coherent and geographically meaningful identification of contiguous hotspot clusters.	Section 2.2.3 (Methods)
**6. Strength of Conclusions** - Please make a clearer distinction between statistical significance, effect size, and predictive performance.	We have revised the manuscript to explicitly distinguish between these concepts. We now clearly note that while MCV1 coverage shows a statistically significant association ($p < 0.001$), the effect size ($\beta = -0.378$) is modest, and the predictive performance is very low (marginal $R^2 = 0.022$, AUC = 0.519). We have added careful language to ensure readers	Results section, Discussion section

	understand that statistical significance does not translate to strong predictive or practical significance.	
**7. Surveillance Data Limitations** - Please discuss the possible impact of low laboratory confirmation rates on spatial and temporal results more thoroughly.	We have expanded the discussion of surveillance limitations in both the main Discussion and the dedicated Limitations subsection. We now note that only 14.4% of cases were laboratory-confirmed, which may introduce misclassification bias. However, we also clarify that a sensitivity analysis using only laboratory-confirmed cases showed similar spatial hotspot patterns (results not shown, but noted in the text).	Discussion section (Limitations subsection)
**8. English Language Editing** - The manuscript would benefit from careful English-language editing to address grammatical errors, awkward sentence structures, and punctuation issues.	We have carefully revised the entire manuscript for language and grammar. Specific corrections include: "coverage is still lower than ≥95% percent" corrected to "coverage remains below the ≥95% threshold"; restructuring of overly long sentences in the Introduction and Discussion; and correction of punctuation errors throughout. We have also ensured consistent use of terminology as recommended.	Throughout manuscript
**9. Avoid Overly Rhetorical Expressions** - Please avoid expressions like Nigeria as "a tale of two countries" in the Conclusion.	We have revised the Conclusion to remove this rhetorical expression. The revised conclusion now provides a more direct scientific account of the north-south differences and the distinct strategic responses required for each region.	Conclusion section
**10. Consistent Terminology** - Ensure terms like case ratio, incidence, burden, transmission, and risk are used consistently throughout.	We have carefully reviewed the manuscript to ensure consistent use of terminology throughout. Specifically: we use "case ratio" for our metric per 100,000 live births; "burden" for cumulative cases; "transmission" for the process of disease spread; and "risk" for areas with high vulnerability. Each term is used precisely and consistently.	

We sincerely thank the editors and reviewers for their thoughtful and constructive comments, which have significantly improved the quality and clarity of our manuscript. We believe all concerns have been adequately addressed and that the revised manuscript is substantially strengthened.

Corresponding Author: Polycarp Dauda Madaki
Email: polycarp.madaki@tuks.co.za
