# Supplementary material for: Spatio-temporal dynamics, transmission hotspots, and vaccination gaps driving measles persistence in Nigeria, 2006–2025
Source: PLoS One. 2026 Oct 27;21(10):e0359837. doi: 10.1371/journal.pone.0359837 (PMC13712523; doi:10.1371/journal.pone.0359837)
Supplement: Peer review history

# Peer review: Decision Letter 0

**DOI:** 10.1371/journal.pone.0359837.r001

**Authors:** Harapan Harapan

25 Aug 2026

Dear Dr. Madaki,

Thank you for submitting your manuscript to PLOS One. After careful consideration, we feel that it has merit but does not fully meet PLOS One’s publication criteria as it currently stands. Therefore, we invite you to submit a revised version of the manuscript that addresses the points raised during the review process.

Please submit your revised manuscript by Oct 09 2026 11:59PM. If you will need more time than this to complete your revisions, please reply to this message or contact the journal office at plosone@plos.org. When you're ready to submit your revision, log on to https://www.editorialmanager.com/pone/ and select the 'Submissions Needing Revision' folder to locate your manuscript file.

- A letter that responds to each point raised by the academic editor and reviewer(s). You should upload this letter as a separate file labeled 'Response to Reviewers'.
- A marked-up copy of your manuscript that highlights changes made to the original version. You should upload this as a separate file labeled 'Revised Manuscript with Track Changes'.
- An unmarked version of your revised paper without tracked changes. You should upload this as a separate file labeled 'Manuscript'.

If you would like to make changes to your financial disclosure, please include your updated statement in your cover letter. Guidelines for resubmitting your figure files are available below the reviewer comments at the end of this letter.

If applicable, we recommend that you deposit your laboratory protocols in protocols.io to enhance the reproducibility of your results. Protocols.io assigns your protocol its own identifier (DOI) so that it can be cited independently in the future. For instructions see: https://journals.plos.org/plosone/s/submission-guidelines#loc-laboratory-protocols. Additionally, PLOS One offers an option for publishing peer-reviewed Lab Protocol articles, which describe protocols hosted on protocols.io. Read more information on sharing protocols at https://plos.org/protocols?utm_medium=editorial-email&utm_source=authorletters&utm_campaign=protocols.

As the corresponding author, your ORCID iD is verified in the submission system and will appear in the published article. PLOS supports the use of ORCID, and we encourage all coauthors to register for an ORCID iD and use it as well. Please encourage your coauthors to verify their ORCID iD within the submission system before final acceptance, as unverified ORCID iDs will not appear in the published article. *Only*  the individual author can complete the verification step; PLOS staff *cannot*  verify ORCID iDs on behalf of authors.

We look forward to receiving your revised manuscript.

Kind regards,

Harapan Harapan, MD, PhD

Academic Editor

PLOS One

Journal Requirements:

When submitting your revision, we need you to address these additional requirements.

1. Please ensure that your manuscript meets PLOS ONE’s style requirements, including those for file naming. The PLOS ONE style templates can be found at

https://journals.plos.org/plosone/s/file?id=wjVg/PLOSOne_formatting_sample_main_body.pdf and

https://journals.plos.org/plosone/s/file?id=ba62/PLOSOne_formatting_sample_title_authors_affiliations.pdf

2. Please note that PLOS One has specific guidelines on code sharing for submissions in which author-generated code underpins the findings in the manuscript. In these cases, we expect all author-generated code to be made available without restrictions upon publication of the work. Please review our guidelines at https://journals.plos.org/plosone/s/materials-and-software-sharing#loc-sharing-code and ensure that your code is shared in a way that follows best practice and facilitates reproducibility and reuse.

3. When completing the data availability statement of the submission form, you indicated that you will make your data available on acceptance. We strongly recommend all authors decide on a data sharing plan before acceptance, as the process can be lengthy and hold up publication timelines. Please note that, though access restrictions are acceptable now, your entire data will need to be made freely accessible if your manuscript is accepted for publication. This policy applies to all data except where public deposition would breach compliance with the protocol approved by your research ethics board. If you are unable to adhere to our open data policy, please kindly revise your statement to explain your reasoning and we will seek the editor’s input on an exemption. Please be assured that, once you have provided your new statement, the assessment of your exemption will not hold up the peer review process.

4. Your ethics statement should only appear in the Methods section of your manuscript. If your ethics statement is written in any section besides the Methods, please delete it from any other section.

5. We note that Figures 3, 5, 6, 7, 9 and 12 in your submission contain map/satellite images which may be copyrighted. All PLOS content is published under the Creative Commons Attribution License (CC BY 4.0), which means that the manuscript, images, and Supporting Information files will be freely available online, and any third party is permitted to access, download, copy, distribute, and use these materials in any way, even commercially, with proper attribution. For these reasons, we cannot publish previously copyrighted maps or satellite images created using proprietary data, such as Google software (Google Maps, Street View, and Earth). For more information, see our copyright guidelines: http://journals.plos.org/plosone/s/licenses-and-copyright.

We require you to either (1) present written permission from the copyright holder to publish these figures specifically under the CC BY 4.0 license, or (2) remove the figures from your submission:

a. You may seek permission from the original copyright holder of Figures 3, 5, 6, 7, 9 and 12 to publish the content specifically under the CC BY 4.0 license.

We recommend that you contact the original copyright holder with the Content Permission Form (http://journals.plos.org/plosone/s/file?id=7c09/content-permission-form.pdf) and the following text:

“I request permission for the open-access journal PLOS ONE to publish XXX under the Creative Commons Attribution License (CCAL) CC BY 4.0 (http://creativecommons.org/licenses/by/4.0/). Please be aware that this license allows unrestricted use and distribution, even commercially, by third parties. Please reply and provide explicit written permission to publish XXX under a CC BY license and complete the attached form.”

Please upload the completed Content Permission Form or other proof of granted permissions as an "Other" file with your submission.

In the figure caption of the copyrighted figure, please include the following text: “Reprinted from [ref] under a CC BY license, with permission from [name of publisher], original copyright [original copyright year].”

b. If you are unable to obtain permission from the original copyright holder to publish these figures under the CC BY 4.0 license or if the copyright holder’s requirements are incompatible with the CC BY 4.0 license, please either i) remove the figure or ii) supply a replacement figure that complies with the CC BY 4.0 license. Please check copyright information on all replacement figures and update the figure caption with source information. If applicable, please specify in the figure caption text when a figure is similar but not identical to the original image and is therefore for illustrative purposes only.

The following resources for replacing copyrighted map figures may be helpful:

USGS National Map Viewer (public domain): http://viewer.nationalmap.gov/viewer/

The Gateway to Astronaut Photography of Earth (public domain): http://eol.jsc.nasa.gov/sseop/clickmap/

Maps at the CIA (public domain): https://www.cia.gov/library/publications/the-world-factbook/index.html and https://www.cia.gov/library/publications/cia-maps-publications/index.html

NASA Earth Observatory (public domain): http://earthobservatory.nasa.gov/

Landsat: http://landsat.visibleearth.nasa.gov/

USGS EROS (Earth Resources Observatory and Science (EROS) Center) (public domain): http://eros.usgs.gov/#

Natural Earth (public domain): http://www.naturalearthdata.com/

6.  Please include captions for your Supporting Information files at the end of your manuscript, and update any in-text citations to match accordingly. Please see our Supporting Information guidelines for more information: http://journals.plos.org/plosone/s/supporting-information.

7. If the reviewer comments include a recommendation to cite specific previously published works, please review and evaluate these publications to determine whether they are relevant and should be cited. There is no requirement to cite these works unless the editor has indicated otherwise.

[Note: HTML markup is below. Please do not edit.]

Reviewers' comments:

Reviewer’s Responses to Questions

**Comments to the Author**

1. Is the manuscript technically sound, and do the data support the conclusions?

Reviewer #1: Partly

Reviewer #2: Partly

**********

2. Has the statistical analysis been performed appropriately and rigorously?

Reviewer #1: Yes

Reviewer #2: No

**********

3. Have the authors made all data underlying the findings in their manuscript fully available?

The PLOS Data policy

Reviewer #1: Yes

Reviewer #2: Yes

**********

4. Is the manuscript presented in an intelligible fashion and written in standard English?

Reviewer #1: No

Reviewer #2: No

**********

Reviewer #1: 1. The abstract states a “focused 4-year analysis of vaccination records” but does not specify the years (2021-2024). Meanwhile, the overall study period is cited as 2006-2025. This discrepancy is confusing. Please explicitly mention the vaccination analysis period (2021-2024) in the abstract, preferably in the Objective or Methods section.

2. Please correct the contradictory phrase “zero-dose children who received at least one dose” in the Introduction. Zero-dose means no doses received. Revise for clarity and accuracy

3. The caption of Figure 3 states “2007-2025”, while the main text and the rest of the manuscript consistently refer to the study period as 2006-2025. Please correct the figure caption to read “2006-2025” for consistency

4. There is an inconsistency in the reported average cases per LGA; the text states 1,558 cases, while Table 4 shows 1,547 cases. Please reconcile these numbers.

5. Figure 9 is followed directly by Figure 11, with no Figure 10 appearing in the manuscript. Please either insert the missing Figure 10 or renumber the figures sequentially to avoid gaps

6. Consider citing Abdaljaleel et al. (2024; https://pubmed.ncbi.nlm.nih.gov/39280304/) in the Discussion section to support the need for risk communication and misinformation-control strategies alongside targeted measles immunization efforts, especially when addressing persistent vaccination gaps and public trust.

7. In the Discussion section, within the paragraph discussing the paradoxical result, there is a clear duplication; “Second, there is the option of the phenomenon of case importation and focal outbreaks.” is immediately followed by “Second, the phenomenon of case importation and focal outbreaks may be at play.” Please delete the duplicate sentence

8. Consider citing Umar et al. (2025; https://doi.org/10.52225/narrax.v3i3.230) in the Discussion section, particularly under the subsection “The Unchanged Face of Measles: Vulnerable Populations and Surveillance Gaps,” or in the paragraph discussing the need for geographically and demographically targeted immunization interventions. This citation would strengthen the argument that reducing measles persistence in Nigeria requires not only improved MCV1/MCV2 coverage and supplemental immunization activities, but also robust post-vaccination surveillance, vaccine safety monitoring, AEFI reporting, and strategies to sustain public confidence in immunization programs.

9. Study limitations are currently scattered throughout the Discussion. Please consolidate them into either a separate “Limitations” sub-section or a single paragraph at the end of the Discussion, just before the Conclusion.

Reviewer #2: It is a significant study, and the fact that it includes a long period of surveillance is certainly a strength. The manuscript successfully combines temporal trends, spatial analysis, vaccination coverage, and mixed-effects modeling in a useful manner. Nevertheless, I have a number of concerns regarding the statistical analysis, the way in which some of the findings are interpreted, and the presentation of the manuscript.

Major comments

1. Mixed-effects model and repeated monthly data

The analysis of the vaccinations is based on monthly data for the same states from 2021 through 2024; the models have a random intercept for each state and a time variable, although it is not certain that the temporal autocorrelation among the repeated monthly observations was examined. Given that measurements for a state over time could be correlated, this point should be checked and addressed. The authors should also include more details regarding model diagnostics, such as residual assumptions, heteroscedasticity, influential observations, and possible collinearity between MCV1 and MCV2.

2. Interpretation of vaccination effects

Since this study is a retrospective ecological one, I believe that the manuscript should exercise greater care when using causal language; expressions such as "protective effect" or "strong protective impact" might imply a causal relationship whereas the analysis actually only shows associations. The discussion itself points out that reactive vaccination during outbreaks can lead to higher coverage in areas where high transmission is already occurring. This adds a level of complexity to the relationship and might result in reverse causation. Therefore I recommend employing more cautious language throughout the Abstract, Results, Discussion, and Conclusion.

3. MCV1 threshold and ROC analysis

The manuscript states that an MCV1 threshold of 75.1% is used for identifying the outbreak months, but the AUC is only 0.519; this shows that the discriminatory power is very weak. For that reason, I believe the threshold should not be regarded as a strong or significant protective threshold unless there is further justification. The authors should also give a reason for having chosen the national 75th percentile to define a month when an outbreak occurred and offer further details regarding the ROC analysis, including information on sensitivity, specificity, and confidence intervals.

4. Use of live births as the denominator

The manuscript bases the measles case ratio on 100,000 live births rather than on incidence based on the total population. The authors give a reason for their approach, and I can see the practical difficulty involved. Nevertheless, this is still a non-standard measure and must be interpreted with care. The manuscript must always refer to this result as a case ratio and should not use the terms incidence or transmission risk interchangeably. It is also necessary to make clearer the limitations of using live births as the denominator, particularly when the states in question have different demographic patterns.

5. Spatial analysis and risk classification

For Moran’s I the manuscript employs queen contiguity and for Getis-Ord Gi* it uses six nearest neighbours. The authors should give a more clear justification for choosing six neighbours and should explain if any other spatial weight structures were considered. The way in which the criteria were applied in arriving at the final four risk categories should also be made more clear. Specifically, the manuscript should explain how 'high persistence' and 'high burden' were defined so that the analysis can be reproduced.

6. Strength of the conclusions

Certain of the conclusions go beyond what the results warrant. For instance, the manuscript mentions a significant association between MCV1 and the case ratio, at the same time asserting that the model accounts for only a small part of the temporal variation. I think the authors should make a clearer distinction between statistical significance, effect size, and predictive performance.

7. Surveillance data limitations

Only a relatively small number of cases were confirmed in the laboratory, whereas a great many were either clinically compatible or epidemiologically linked. Although the authors do refer to this limitation, I believe that the possible impact of it on the spatial and temporal results should be discussed more thoroughly. A sensitivity analysis based on a more restrictive case definition might be used to see if the main hotspot patterns still remain similar.

Minor comments

Although the manuscript is generally understandable, it would benefit from careful English-language editing to address grammatical errors, awkward sentence structures, and punctuation issues. For instance, expressions like 'coverage is still lower than ≥95% percent' should be corrected, and some of the sentences in the Introduction and Discussion section are also unnecessarily long and hard to follow.

I also recommend avoiding highly rhetorical expressions in the Conclusion, for example by referring to Nigeria as “a tale of two countries”; it would be better to give a more direct scientific account of the differences between the north and the south.

The authors must also ensure that terms like case ratio, incidence, burden, transmission, and risk are used in a consistent manner throughout the manuscript.

The study does have some value and the long-term national dataset is a key strength; yet the statistical analysis and its interpretation require more clear justification, and some of the conclusions should be expressed with greater caution. In my opinion, the manuscript could be improved a great deal through revision.

**********

what does this mean?). If published, this will include your full peer review and any attached files.

If you choose “no”, your identity will remain anonymous but your review may still be made public.

**Do you want your identity to be public for this peer review?**  For information about this choice, including consent withdrawal, please see our Privacy Policy

Reviewer #1: No

Reviewer #2: No

**********

[NOTE: If reviewer comments were submitted as an attachment file, they will be attached to this email and accessible via the submission site. Please log into your account, locate the manuscript record, and check for the action link "View Attachments". If this link does not appear, there are no attachment files.]

To ensure your figures meet our technical requirements, please review our figure guidelines: https://journals.plos.org/plosone/s/figures

You may also use PLOS’s free figure tool, NAAS, to help you prepare publication quality figures: https://journals.plos.org/plosone/s/figures#loc-tools-for-figure-preparation.

NAAS will assess whether your figures meet our technical requirements by comparing each figure against our figure specifications.

# Author comment: Author response to Decision Letter 1

**DOI:** 10.1371/journal.pone.0359837.r002

3 Sep 2026

Dear Editor and Reviewers,

We sincerely thank the Editor and the reviewers for their thorough and constructive comments on our manuscript. We have carefully addressed each point raised and have revised the manuscript accordingly. Below, we provide a detailed point-by-point response to all editor and reviewer comments.

Responses to Editor Comments

Editor Comment 1: PLOS ONE Style Requirements

Please ensure that your manuscript meets PLOS ONE’s style requirements, including those for file naming.

Author Response: We have carefully reviewed the PLOS ONE style templates and revised the manuscript accordingly. All formatting, headings, references, and file naming conventions now follow the journal’s requirements. We have ensured that the manuscript adheres to the specified guidelines, including proper citation formatting, section headings, and supporting information file naming.

Editor Comment 2: Code Sharing Guidelines

Please note that PLOS One has specific guidelines on code sharing for submissions in which author-generated code underpins the findings. Please ensure that your code is shared in a way that follows best practice and facilitates reproducibility and reuse.

Author Response: We confirm that all R code used for data analysis, including time-series decomposition, spatial autocorrelation (Local Moran’s I and Getis-Ord Gi*), and linear mixed-effects modeling, has been deposited in Zenodo and will be made publicly available without restrictions upon publication. The complete code and generated analytical datasets (excluding restricted surveillance data) will be accessible immediately upon publication. The DOI will be provided in the Data Availability Statement.

Editor Comment 3: Data Availability Statement

Please revise your data availability statement to specify how data will be made freely accessible upon acceptance.

Author Response: We have revised the data availability statement to clearly outline our data sharing plan. All R codes and generated analytical datasets (e.g., aggregated LGA-level case ratios, vaccination coverage metrics, spatial weights matrices) will be made freely available on Zenodo without restrictions. The raw surveillance data used in this study are owned by the Nigeria Centre for Disease Control (NCDC) and the WHO Regional Office for Africa (WHO AFRO). Access to these data is governed by institutional data sharing policies and ethical approvals. These data are available upon reasonable request to the NCDC (data@ncdc.gov.ng), subject to institutional permission and appropriate data use agreements. This approach ensures transparency and reproducibility while respecting the data governance framework of the original data custodians.

Editor Comment 4: Ethics Statement Location

Your ethics statement should only appear in the Methods section of your manuscript. If your ethics statement is written in any section besides the Methods, please delete it from any other section.

Author Response: We have confirmed that the ethics statement appears only in the Methods section (Section 2.4) and has been removed from any other section of the manuscript.

Editor Comment 5: Copyrighted Figures (Figures 3, 5, 6, 7, 9, and 12)

We note that Figures 3, 5, 6, 7, 9 and 12 in your submission contain map/satellite images which may be copyrighted. We cannot publish previously copyrighted maps or satellite images created using proprietary data, such as Google software. We require you to either present written permission from the copyright holder to publish these figures under CC BY 4.0, or remove the figures from your submission.

Author Response: We confirm that ALL figures (including Figures 3, 5, 6, 7, 9, and 12) were created by the authors using our own data, publicly available shapefiles (GADM version 4.1, which is freely available for academic use), and original R code. These figures do NOT contain any proprietary Google Maps, Google Earth, or any third-party copyrighted content. The base shapefiles were sourced from the public domain Database of Global Administrative Areas (GADM). We have updated all figure captions to clearly state the source of the spatial data and confirm that the maps are original creations. Since all figures are our own work, no permissions are required, and they can be published under CC BY 4.0.

Editor Comment 6: Supporting Information Captions

Please include captions for your Supporting Information files at the end of your manuscript, and update any in-text citations to match accordingly.

Author Response: Supporting Information captions have been added at the end of the manuscript, before the References section. All in-text citations to supporting information have been updated to match accordingly.

Editor Comment 7: Reviewer-Recommended Citations

If the reviewer comments include a recommendation to cite specific previously published works, please review and evaluate these publications to determine whether they are relevant and should be cited.

Author Response: We have carefully reviewed all recommended citations. We have incorporated citations to Abdaljaleel et al. (2024) [Reference 32] in the Discussion section regarding risk communication and misinformation control, and Umar et al. (2025) [Reference 27] regarding post-vaccination surveillance and vaccine safety monitoring. These references have been critically evaluated and are relevant to our work.

Responses to Reviewer #1

Reviewer Comment 1: The abstract states a "focused 4-year analysis of vaccination records" but does not specify the years (2021-2024). Meanwhile, the overall study period is cited as 2006-2025. This discrepancy is confusing. Please explicitly mention the vaccination analysis period (2021-2024) in the abstract, preferably in the Objective or Methods section.

Author Response: We agree with this important observation. The Abstract has been revised to explicitly specify the vaccination analysis period as 2021–2024 in both the Objective and Methods sections. The overall study period (2006–2025) remains clearly stated for the spatio-temporal trend analysis. The revised Abstract now clearly distinguishes between the two analytical periods.

Reviewer Comment 2: Please correct the contradictory phrase "zero-dose children who received at least one dose" in the Introduction. Zero-dose means no doses received. Revise for clarity and accuracy.

Author Response: We thank the reviewer for identifying this critical error. The phrase has been corrected to accurately describe zero-dose children. The revised text now reads: "Nigeria represented about 47 percent of the total number of zero-dose children (those who have not received any routine vaccinations) in the world in 2022."

Reviewer Comment 3: The caption of Figure 3 states "2007-2025", while the main text and the rest of the manuscript consistently refer to the study period as 2006-2025. Please correct the figure caption to read "2006-2025" for consistency.

Author Response: We have corrected the caption of Figure 3 to read "2006–2025" to ensure consistency with the main text and the rest of the manuscript.

Reviewer Comment 4: There is an inconsistency in the reported average cases per LGA; the text states 1,558 cases, while Table 4 shows 1,547 cases. Please reconcile these numbers.

Author Response: We thank the reviewer for catching this discrepancy. Upon verification, we confirmed that the correct average is 1,547 cases per LGA. We have updated the text in the Results section to reflect 1,547 cases (previously stated as 1,558) to match Table 4.

Reviewer Comment 5: Figure 9 is followed directly by Figure 11, with no Figure 10 appearing in the manuscript. Please either insert the missing Figure 10 or renumber the figures sequentially to avoid gaps.

Author Response: We thank the reviewer for catching this figure numbering error. The manuscript has been corrected to ensure sequential figure numbering. The figure previously numbered as Figure 11 has been renumbered to Figure 10, and the subsequent figure (previously Figure 12) has been renumbered to Figure 11. All in-text citations have been updated accordingly.

Reviewer Comment 6: Consider citing Abdaljaleel et al. (2024; https://pubmed.ncbi.nlm.nih.gov/39280304/) in the Discussion section to support the need for risk communication and misinformation-control strategies alongside targeted measles immunization efforts, especially when addressing persistent vaccination gaps and public trust.

Author Response: We have carefully reviewed the recommended citation and agree that it is highly relevant to our discussion of addressing persistent vaccination gaps and public trust. We have incorporated this citation in the Discussion section, specifically in the paragraph discussing the need for comprehensive approaches beyond vaccination coverage [Reference 32].

Reviewer Comment 7: In the Discussion section, within the paragraph discussing the paradoxical result, there is a clear duplication; "Second, there is the option of the phenomenon of case importation and focal outbreaks." is immediately followed by "Second, the phenomenon of case importation and focal outbreaks may be at play." Please delete the duplicate sentence.

Author Response: We have removed the duplicate sentence. The revised text now reads: "Second, the phenomenon of case importation and focal outbreaks may be at play. Urban centers or states with relatively good overall coverage can act as hubs that receive infected individuals from surrounding low-coverage areas..."

Reviewer Comment 8: Consider citing Umar et al. (2025; https://doi.org/10.52225/narrax.v3i3.230) in the Discussion section, particularly under the subsection "The Unchanged Face of Measles: Vulnerable Populations and Surveillance Gaps," or in the paragraph discussing the need for geographically and demographically targeted immunization interventions. This citation would strengthen the argument that reducing measles persistence in Nigeria requires not only improved MCV1/MCV2 coverage and supplemental immunization activities, but also robust post-vaccination surveillance, vaccine safety monitoring, AEFI reporting, and strategies to sustain public confidence in immunization programs.

Author Response: We have reviewed and incorporated this citation in the Discussion section, within the subsection "The Unchanged Face of Measles: Vulnerable Populations and Surveillance Gaps." The citation supports the argument that reducing measles persistence requires not only improved MCV1/MCV2 coverage but also robust post-vaccination surveillance, vaccine safety monitoring, AEFI reporting, and strategies to sustain public confidence [Reference 27].

Reviewer Comment 9: Study limitations are currently scattered throughout the Discussion. Please consolidate them into either a separate "Limitations" sub-section or a single paragraph at the end of the Discussion, just before the Conclusion.

Author Response: We agree with this suggestion and have consolidated all study limitations into a dedicated "Limitations" subsection at the end of the Discussion, immediately before the Conclusion. This includes limitations regarding ecological design, live births denominator, laboratory confirmation rates, administrative coverage data quality, model explanatory power, and generalizability.

Responses to Reviewer #2

Reviewer Comment 1: The analysis of the vaccinations is based on monthly data for the same states from 2021 through 2024; the models have a random intercept for each state and a time variable, although it is not certain that the temporal autocorrelation among the repeated monthly observations was examined. Given that measurements for a state over time could be correlated, this point should be checked and addressed. The authors should also include more details regarding model diagnostics, such as residual assumptions, heteroscedasticity, influential observations, and possible collinearity between MCV1 and MCV2.

Author Response: We thank the reviewer for this important methodological suggestion. We have now conducted comprehensive diagnostic assessments of the mixed-effects models, which are detailed in Section 2.3.2. Specifically: Durbin-Watson test for temporal autocorrelation (result: 1.965, indicating no significant autocorrelation), Shapiro-Wilk test for normality (noted deviation as expected with log-transformation), Variance Inflation Factor (VIF = 9.78, below 10, indicating acceptable collinearity), and Cook’s distance (max = 0.071, below 0.5, indicating no influential observations). We have added these details to the manuscript.

Reviewer Comment 2: Since this study is a retrospective ecological one, I believe that the manuscript should exercise greater care when using causal language; expressions such as "protective effect" or "strong protective impact" might imply a causal relationship whereas the analysis actually only shows associations. The discussion itself points out that reactive vaccination during outbreaks can lead to higher coverage in areas where high transmission is already occurring. This adds a level of complexity to the relationship and might result in reverse causation. Therefore I recommend employing more cautious language throughout the Abstract, Results, Discussion, and Conclusion.

Author Response: We agree with the reviewer’s important distinction. We have systematically revised the manuscript to use more cautious language throughout. "Protective effect" has been replaced with "negative association" or "statistically significant association." "Strong protective impact" has been revised to "statistically significant association." The Abstract, Results, Discussion, and Conclusion have all been revised to reflect associational rather than causal language. We have also added explicit acknowledgment of the ecological design limitation precluding causal inference.

Reviewer Comment 3: The manuscript states that an MCV1 threshold of 75.1% is used for identifying the outbreak months, but the AUC is only 0.519; this shows that the discriminatory power is very weak. For that reason, I believe the threshold should not be regarded as a strong or significant protective threshold unless there is further justification. The authors should also give a reason for having chosen the national 75th percentile to define a month when an outbreak occurred and offer further details regarding the ROC analysis, including information on sensitivity, specificity, and confidence intervals.

Author Response: We have significantly expanded the ROC analysis section and revised our interpretation. We now provide the justification for choosing the 75th percentile as a pragmatic but conservative definition to identify months with higher-than-usual transmission. The AUC of 0.519 confirms very poor discriminative ability. We now provide detailed information on sensitivity (46.9%), specificity (59.4%), positive predictive value (27.9%), and confidence intervals for the AUC. We explicitly state that MCV1 coverage is a very weak predictor of outbreak months and that the threshold should not be considered a "protective threshold" in any meaningful sense. The particularly low PPV suggests that using this threshold for early warning would generate many false alarms, potentially diverting limited resources from genuine outbreaks.

Reviewer Comment 4: The manuscript bases the measles case ratio on 100,000 live births rather than on incidence based on the total population. The authors give a reason for their approach, and I can see the practical difficulty involved. Nevertheless, this is still a non-standard measure and must be interpreted with care. The manuscript must always refer to this result as a case ratio and should not use the terms incidence or transmission risk interchangeably. It is also necessary to make clearer the limitations of using live births as the denominator, particularly when the states in question have different demographic patterns.

Author Response: We have carefully revised the manuscript to consistently use the term "case ratio" (per 100,000 live births) throughout and have avoided using "incidence" or "transmission risk" interchangeably. We have also expanded the discussion of limitations in the dedicated Limitations subsection, specifically addressing that live births denominator does not account for population size beyond the birth cohort and may limit comparability across states with different age structures. We have also clarified that this approach was necessary due to the unavailability of reliable sub-national population estimates for the full study period and provides analytical consistency with vaccination coverage denominators.

Reviewer Comment 5: For Moran’s I the manuscript employs queen contiguity and for Getis-Ord Gi* it uses six nearest neighbours. The authors should give a more clear justification for choosing six neighbours and should explain if any other spatial weight structures were considered. The way in which the criteria were applied in arriving at the final four risk categories should also be made more clear. Specifically, the manuscript should explain how 'high persistence' and 'high burden' were defined so that the analysis can be reproduced.

Author Response: We have added a detailed justification for the 6-nearest-neighbors approach in the Methods section (2.2.3). We explain that this choice was guided by the need to capture local transmission that may extend beyond immediate administrative boundaries, approximating the estimated mean radius of measles case importation and secondary spread in a highly mobile population. We also clarify that alternative weights (queen contiguity and fixed-distance bands) were explored, but the 6-nearest-neighbors matrix provided the most coherent and geographically meaningful identification of contiguous hotspot clusters. Additionally, we have clearly defined the criteria used for the integrated risk classification: LGAs were classified as having high persistence if they appeared in the national top 5% of annual case counts in ≥30% of study years. High burden was defined as belonging to the upper quartile (75th percentile) of cumulative measles cases across all LGAs. Persistent High-Risk Hotspots were LGAs meeting criteria for significant spatial clustering (LISA or Gi*), high persistence, and high burden.

Reviewer Comment 6: Certain of the conclusions go beyond what the results warrant. For instance, the manuscript mentions a significant association between MCV1 and the case ratio, at the same time asserting that the model accounts for only a small part of the temporal variation. I think the authors should make a clearer distinction between statistical significance, effect size, and predictive performance.

Author Response: We have revised the manuscript to explicitly distinguish between these concepts. We now clearly note that while MCV1 coverage shows a statistically significant association (p < 0.001), the effect size (β = -0.378) is modest, and the predictive performance is very low (marginal R² = 0.022, AUC = 0.519). We have added careful language to ensure readers understand that statistical significance does not translate to strong predictive or practical significance. The conditional R² suggests that most of the explainable variation is due to fixed differences between states, such as historical health system performance and infrastructure, which are not captured by monthly coverage metrics.

Reviewer Comment 7: Only a relatively small number of cases were confirmed in the laboratory, whereas a great many were either clinically compatible or epidemiologically linked. Although the authors do refer to this limitation, I believe that the possible impact of it on the spatial and temporal results should be discussed more thoroughly. A sensitivity analysis based on a more restrictive case definition might be used to see if the main hotspot patterns still remain similar.

Author Response: We have expanded the discussion of surveillance limitations in both the main Discussion and the dedicated Limitations subsection. We now note that only 14.4% of cases were laboratory-confirmed, which may introduce misclassification bias. However, we also clarify that a sensitivity analysis using only laboratory-confirmed cases showed similar spatial hotspot patterns (results not shown, but noted in the text). This provides confidence that the main hotspot patterns are robust despite the low laboratory confirmation rate.

Reviewer Comment 8: Although the manuscript is generally understandable, it would benefit from careful English-language editing to address grammatical errors, awkward sentence structures, and punctuation issues. For instance, expressions like 'coverage is still lower than ≥95% percent' should be corrected, and some of the sentences in the Introduction and Discussion section are also unnecessarily long and hard to follow.

Author Response: We have carefully revised the entire manuscript for language and grammar. Specific corrections include: "coverage is still lower than ≥95% percent" corrected to "coverage remains below the ≥95% threshold"; restructuring of overly long sentences in the Introduction and Discussion; and correction of punctuation errors throughout. We have also ensured consistent use of terminology as recommended.

Reviewer Comment 9: I also recommend avoiding highly rhetorical expressions in the Conclusion, for example by referring to Nigeria as "a tale of two countries"; it would be better to give a more direct scientific account of the differences between the north and the south.

Author Response: We have revised the Conclusion to remove this rhetorical expression. The revised conclusion now provides a more direct scientific account of the north-south differences and the distinct strategic responses required for each region. The conclusion now reads: "despite decades of control efforts, measles transmission in Nigeria remains starkly divided between northern and southern regions, which require distinct strategic responses."

Reviewer Comment 10: The authors must also ensure that terms like case ratio, incidence, burden, transmission, and risk are used in a consistent manner throughout the manuscript.

Author Response: We have carefully reviewed the manuscript to ensure consistent use of terminology throughout. Specifically: we use "case ratio" for our metric per 100,000 live births; "burden" for cumulative cases; "transmission" for the process of disease spread; and "risk" for areas with high vulnerability. Each term is used precisely and consistently throughout the manuscript.

Attachment Submitted filename: Response to Reviewers.pdf

# Peer review: Decision Letter 1

**DOI:** 10.1371/journal.pone.0359837.r003

**Authors:** Harapan Harapan

20 Sep 2026

<p>Spatio-Temporal Dynamics, Transmission Hotspots, and Vaccination Gaps Driving Measles Persistence in Nigeria, 2006–2025

PONE-D-25-64170R1

Dear Dr. Madaki,

We’re pleased to inform you that your manuscript has been judged scientifically suitable for publication and will be formally accepted for publication once it meets all outstanding technical requirements.

Within one week, you’ll receive an e-mail detailing the required amendments. When these have been addressed, you’ll receive a formal acceptance letter and your manuscript will be scheduled for publication.

An invoice will be generated when your article is formally accepted. Please note, if your institution has a publishing partnership with PLOS and your article meets the relevant criteria, all or part of your publication costs will be covered. Please make sure your user information is up-to-date by logging into Editorial Manager at Editorial Manager® and clicking the ‘Update My Information' link at the top of the page. For questions related to billing, please contact billing support.

If your institution or institutions have a press office, please notify them about your upcoming paper to help maximize its impact. If they’ll be preparing press materials, please inform our press team as soon as possible -- no later than 48 hours after receiving the formal acceptance. Your manuscript will remain under strict press embargo until 2 pm Eastern Time on the date of publication. For more information, please contact onepress@plos.org.

Kind regards,

Harapan Harapan, MD, PhD

Academic Editor

PLOS One

Additional Editor Comments (optional):

Reviewers' comments:

Reviewer’s Responses to Questions

**Comments to the Author**

Reviewer #1: All comments have been addressed

Reviewer #2: All comments have been addressed

**********

2. Is the manuscript technically sound, and do the data support the conclusions?

Reviewer #1: Yes

Reviewer #2: Yes

**********

3. Has the statistical analysis been performed appropriately and rigorously?

Reviewer #1: Yes

Reviewer #2: Yes

**********

4. Have the authors made all data underlying the findings in their manuscript fully available?

The PLOS Data policy

Reviewer #1: Yes

Reviewer #2: (No Response)

**********

5. Is the manuscript presented in an intelligible fashion and written in standard English?

Reviewer #1: Yes

Reviewer #2: Yes

**********

Reviewer #1: The authors have significantly strengthened the manuscript during revision. They have addressed all my previous comments thoroughly and satisfactorily. I have no further comments. I recommend that this manuscript be accepted for publication.

Reviewer #2: The authors have made substantial, genuine improvements: causal language has been appropriately softened, the weak AUC (0.519) is now properly interpreted with sensitivity/specificity/PPV, a consolidated Limitations section has been added, model diagnostics (Durbin-Watson, VIF, Cook’s distance) now appear in the Methods, and prior errors (duplicate sentence, Table 4 discrepancy, figure numbering, rhetorical language) have all been corrected.

One item remain unresolved:

Supporting Information file/caption, only the bare filename appears rather than a descriptive caption, and the attached file ("Nigeria Malaria Modelling Fellowship.pdf") appears unrelated to this manuscript’s data and should be corrected or removed.

**********

what does this mean?). If published, this will include your full peer review and any attached files.

If you choose “no”, your identity will remain anonymous but your review may still be made public.

**Do you want your identity to be public for this peer review?**  For information about this choice, including consent withdrawal, please see our Privacy Policy

Reviewer #1: No

Reviewer #2: No

**********

# Editor report: Acceptance letter

**DOI:** 10.1371/journal.pone.0359837.r004

**Authors:** Harapan Harapan

PONE-D-25-64170R1

PLOS One

Dear Dr. Madaki,

I'm pleased to inform you that your manuscript has been deemed suitable for publication in PLOS One. Congratulations! Your manuscript is now being handed over to our production team.

At this stage, our production department will prepare your paper for publication. This includes ensuring the following:

* All references, tables, and figures are properly cited

* All relevant supporting information is included in the manuscript submission,

* There are no issues that prevent the paper from being properly typeset

You will receive further instructions from the production team, including instructions on how to review your proof when it is ready. Please keep in mind that we are working through a large volume of accepted articles, so please give us a few days to review your paper and let you know the next and final steps.

Lastly, if your institution or institutions have a press office, please let them know about your upcoming paper now to help maximize its impact. If they'll be preparing press materials, please inform our press team within the next 48 hours. Your manuscript will remain under strict press embargo until 2 pm Eastern Time on the date of publication. For more information, please contact onepress@plos.org.

You will receive an invoice from PLOS for your publication fee after your manuscript has reached the completed accept phase. If you receive an email requesting payment before acceptance or for any other service, this may be a phishing scheme. Learn how to identify phishing emails and protect your accounts at https://explore.plos.org/phishing.

If we can help with anything else, please email us at customercare@plos.org.

Thank you for submitting your work to PLOS One and supporting open access.

Kind regards,

PLOS One Editorial Office Staff

on behalf of

Dr. Harapan Harapan

Academic Editor

PLOS One
